# Global Cross-Talk of Genes of the Mosquito *Aedes aegypti* in Response to Dengue Virus Infection

**DOI:** 10.1371/journal.pntd.0001385

**Published:** 2011-11-15

**Authors:** Susanta K. Behura, Consuelo Gomez-Machorro, Brent W. Harker, Becky deBruyn, Diane D. Lovin, Ryan R. Hemme, Akio Mori, Jeanne Romero-Severson, David W. Severson

**Affiliations:** Eck Institute for Global Health, Department of Biological Sciences, University of Notre Dame, Notre Dame, Indiana, United States of America; University of California Irvine, United States of America

## Abstract

**Background:**

The mosquito *Aedes aegypti* is the primary vector of dengue virus (DENV) infection in humans, and DENV is the most important arbovirus across most of the subtropics and tropics worldwide. The early time periods after infection with DENV define critical cellular processes that determine ultimate success or failure of the virus to establish infection in the mosquito.

**Methods and Results:**

To identify genes involved in these processes, we performed genome-wide transcriptome profiling between susceptible and refractory *A. aegypti* strains at two critical early periods after challenging them with DENV. Genes that responded coordinately to DENV infection in the susceptible strain were largely clustered in one specific expression module, whereas in the refractory strain they were distributed in four distinct modules. The susceptible response module in the global transcriptional network showed significant biased representation with genes related to energy metabolism and DNA replication, whereas the refractory response modules showed biased representation across different metabolism pathway genes including cytochrome P450 and DDT [1,1,1-Trichloro-2,2-bis(4-chlorophenyl) ethane] degradation genes, and genes associated with cell growth and death. A common core set of coordinately expressed genes was observed in both the susceptible and refractory mosquitoes and included genes related to the Wnt (Wnt: wingless [wg] and integration 1 [int1] pathway), MAPK (Mitogen-activated protein kinase), mTOR (mammalian target of rapamycin) and JAK-STAT (Janus Kinase - Signal Transducer and Activator of Transcription) pathways.

**Conclusions:**

Our data revealed extensive transcriptional networks of mosquito genes that are expressed in modular manners in response to DENV infection, and indicated that successfully defending against viral infection requires more elaborate gene networks than hosting the virus. These likely play important roles in the global-cross talk among the mosquito host factors during the critical early DENV infection periods that trigger the appropriate host action in susceptible vs. refractory mosquitoes.

## Author Summary

Dengue virus is primarily transmitted by *Aedes aegypti* mosquitoes. Control of the vector mosquito is the major practice to prevent dengue. However, it is not well known how the virus can infect some mosquito strains but fail to do so with other refractory strains. To address that question, we conducted whole genome microarray based gene expression studies between susceptible and refractory strains of *A. aegypti* to identify gene expression patterns following challenge with dengue virus. Our analysis of the early infection periods reveals that a large number of genes are involved in a highly coordinated manner either to host or defend against the virus. Genes responding to dengue infection were clustered in seven expression modules. Genes associated with susceptibility to infection were largely clustered in one expression module, while those associated with refractoriness were distributed in four distinct modules. A common core set of genes expressed in both susceptible and refractory individuals were clustered in two expression modules. We identified genes and specific pathways that potentially regulate compatible or non-compatible interactions between dengue virus and the mosquito, most notably energy metabolism and DNA replication in the susceptible response in contrast to cell growth and death in the refractory response.

## Introduction

Dengue virus (DENV) represents a significant challenge for global public health where 2.5 billion people are estimated to be at risk of dengue related diseases [1]–[3]. The mosquito *Aedes aegypti* is the primary global vector of DENV. There are no effective vaccines or treatments available, with mosquito control remaining the only viable strategy for disease prevention. The spread of DENV is critically dependent upon successful completion of viral life cycles in the infected mosquito [4]. Understanding the basic mechanisms of how the mosquito successfully transmits DENV is a first requirement towards designing novel genetic control strategies.

Upon mosquito blood feeding on a viremic human, DENV enters the mosquito mid-gut with the blood meal where it must establish an infection in mid-gut epithelial cells, the success of which is required for subsequent completion of the viral life cycle in the mosquito. The intrinsic ability of *A. aegypti* to host the virus is generally referred to as ‘vector competence’. Several anatomical barriers including mid-gut infection barriers (MIB) or mid-gut escape barriers (MEB) contribute to reduced susceptibility of *A. aegypti* mosquitoes to DENV [5]. Though these infection barriers have been demonstrated to be influenced by genes within multiple quantitative trait loci (QTL), the specific genes involved in conferring these infection barriers have not been identified. Natural populations of *A. aegypti* mosquitoes show extensive genetic variation that may account for varying degrees of susceptibility to DENV [5]–[9]. However, the mechanisms and genes that influence vector competence of *A. aegypti* to DENV are not well understood [10].

To better understand the global gene expression pattern of mosquito genes upon DENV infection, we performed genome-wide transcriptome analyses in *A. aegypti* susceptible and refractory strains at two early time points after challenge with DENV. Our data reveals that *A. aegypti* genes show transcriptional responses in a modular manner at early infection periods, wherein groups of genes are expressed in a tightly correlated manner. Our analysis further shows that specific biochemical pathways are enriched among these modularly expressed genes in the susceptible and refractory mosquitoes.

## Materials and Methods

### Ethics statement

This study was performed in accordance with the recommendations in the Guide for the Care and Use of Laboratory Animals of the National Institutes of Health. The animal use protocol was approved by the University of Notre Dame Institutional Animal Care and Use Committee (Study # 11-036).

### Mosquito samples and DENV infections

The *A. aegypti* strains Moyo-S (MS) and Moyo-R (MR) are sub-strains of the Moyo-in-Dry (MD) strain that was collected indoors from Shauri Moyo, near Mombasa, Kenya [11]. Sub-strains were originally selected for *Plasmodium gallinaceum* susceptibility and refractoriness, respectively [12]. Although not selected for DENV susceptibility, the sub-strains also show significant differences in mean DENV serotype 2 (DENV-2) infection rates with ∼20% in one (MR: DENV-2 refractory) and ∼57% in the other (MS: DENV-2 susceptible) [13], while the original MD strain shows natural low DENV infection rates of ∼13% (unpublished data). The D2S3 strain was selected for high oral susceptibility to DENV [14] and shows ∼46% susceptibility to DENV-2 infection under our conditions [13]. Mosquitoes were reared and maintained in an environmental chamber following our standard conditions [13].

Cell culture and mosquito infections were performed as previously described [13]. Briefly, starved females were provided with an artificial infectious blood meal, freshly prepared using defibrinated sheep blood (Colorado Serum Co., CS1122) mixed with (equal volume) a dengue viral suspension. DENV-2 strain JAM1409 was cultured using *Aedes albopictus* C6/36 cells until 80–90% confluence in MEM-EBSS media (HyClone SH3002401) supplemented with 25 mM Hepes buffer, 1 mM sodium pyruvate, 0.025 mg/ml Gentamycin, 1× (0.01 mM) non-essential amino acids and 10% fetal bovine serum (heat inactivated). A 0.1 multiplicity of infection (MOI) was used for infecting the mosquito cells. The MOI refers to the average number of viral particles that infect a single cell, which for our purpose is equal to plaque-forming units (pfu) per cell. The flasks were incubated at 28°C for 7 days after which the viral supernatant was obtained by centrifugation at 1500 rpm for 5 min at 4°C. All DENV infection work was performed in a BSL3 facility.

### Oligonucleotide microarrays

A genome-wide transcriptome analysis was carried out using the NimbleGen oligonucleotide microarray format (www.nimblegen.com). The custom-made high density array (385K format) was designed with 60-mer oligos specific to 16,092 gene transcripts of gene build AaegL1.1 of *A. aegypti* (www.vectorbase.org) [15]. For each transcript, from 1 to 20 different unique probes were designed and used. However, for 99.4% of genes, 20 probes per each gene were used, with the lower number of probes-to-gene being associated with smaller transcripts. The NimbleGen design utilizes the uniqueness of probe sequences as one of the criteria for probe selection to avoid cross-hybridization with non-target genes. The details of the array design, sample description and expression data are available at Gene Expression Omnibus (GEO) under accession number GSE16563. The layout of the probes in the array was made using row and column specifications with the standard 1∶4 format of NimbleGen ArrayScribe.

Total RNA was purified from infected and control samples and forwarded to NimbleGen where labeling and hybridizations were performed following their standard procedures. RNA samples were obtained at 3 hr and 18 hr post blood feeding from DENV-2 infected and control females from each strain. Three independent feeding experiments were performed to obtain three biological replicates of samples for both the strains and the post-infection time points. Fully engorged females were isolated and maintained at 26°C with 84% humidity and in a 16-h light/8-h dark cycle. At each time point, RNA was extracted from 20 females per sample (the blood meal was first removed from each female using a micro syringe needle) using a Qiagen RNAeasy Kit as per manufacturer's instructions. The RNA was quantified by a Nanodrop spectrophotometer and quality of RNA was assessed by a Bioanalyzer.

A total of 15 samples were used for array hybridizations. They included 12 test samples and three control samples. The test samples included three biological replicates for each of the four infected samples (MS-3 hr, MR-3 hr, MS-18 hr and MR-18 hr). A control was prepared for each of the three replicates that consisted of RNA isolated from females fed with uninfected blood meals and pooled across both strains and time points. The two time points were chosen based on our unpublished observations that significant changes in host gene response to DENV infection are already evident within 24 hrs post-infection. The microarray data normalization was performed using the quantile normalization method [16] and the Robust Multichip Average (RMA) algorithm [17]. The Statistical Analysis of Microarray (SAM) software [18] was used to determine significantly (δ = 1.61, fdr = 0.52%) differential expression between test and control samples.

### Prediction of expression modules and gene networking patterns

The expression modules were determined by weighted co-expression analysis of the differentially expressed genes using the Weighted Gene Correlation Network Analysis (WGCNA) program [19]. The ‘topology overlapping’ (TO) within the cluster tree was used in the program to predict the expression modules by a dynamic hybrid cutting method [20]. It is based on pair-wise positive and negative correlations (Pearson correlation matrix) among the differentially expressed genes. The connection strength (connectivity) among the genes was calculated from the absolute value of the matrix raised to a predefined power and genes with similar patterns of connection strengths (or topological overlap) are identified. Using topological overlap values, hierarchical clustering was performed to identify modules of highly interconnected genes. A trait file of input genes was used for analysis in WGCNA. It contained binary numbers (0 or 1) for each gene depending on if the gene was up-regulated or down-regulated in MS or in MR strain, respectively. The WGCNA program was also used to generate the heat maps of gene expression in each module.

Genetic networks of the responsive genes were constructed by using the ‘GeneNet’ package implemented in *R* [21]. The program uses graphical Gaussian models (GGMs) to represent multivariate dependencies of genes based on expression data. The algorithm estimates a partial correlation (pcor) matrix that is then used to calculate shrinkage covariance estimators of gene expression [22]. Once the shrinkage estimators of pcor values are generated, the program performs GGM selection by multiple testing of false discovery rates that are used to define the nodes and edges of the association network by an empirical Bayes approach [23]. Although graphical GGMs are generally applied to independent and identically distributed data, GeneNet incorporates provisions for small scale datasets, where the observation time points may be unequally spaced. We used the expression data at 3 hr and 18 hr time points for the 2,455 responsive genes to estimate partial correlations. The input data for this estimation was generated by use of the ‘longitudinal’ program included in the GeneNet package. The graphical view of the networks was either created by using Graphviz 2.18 (http://www.graphviz.org/) or the pair-wise pcor values were extracted for further analyses. The interacting gene pairs identified from GeneNet program were obtained in a tabular form and were compared with networking genes predicted by WGCNA to determine the inter- and intra-modularly interacting genes.

### Pathway annotation of responsive genes

In order to understand the functional characteristics of the modular gene expression patterns, we determined if genes of specific pathway(s) are over-represented in the modularly expressed genes. The *A. aegypti* pathway genes were obtained from KEGG (Koyota Encyclopedia of Genes and Genomes, Japan; http://www.genome.jp/kegg/) in October, 2008. The biased representation of pathway genes was determined by mapping KEGG pathway genes annotated for *A. aegypti* (http://www.genome.jp/kegg-bin/show_organism?org=aag). The observed numbers of pathway genes in each of the expression modules (predicted from array data) were counted. The cumulative value of gene counts representing a KEGG module was obtained by determining the total number of genes representing each pathway included in the KEGG module. We assumed a null hypothesis where the annotated KEGG pathways had a non-biased representation to the expression modules identified from our array data. Under this assumption, the expected number of genes representing each KEGG module relative to each of our predicted expression modules was the mean number of genes per KEGG module. A test of goodness-of-fit was conducted from the observed and expected gene counts for each expression module using Pearson's Chi-square method.

### qRT-PCR validation of responsive genes

Expression levels of randomly selected responsive genes (n = 5) from the microarray analyses were validated using SYBR Green dye technology (Applied Biosystems) by quantitative real-time PCR (qRT-PCR). The qRT-PCR assays were performed with RNAs from MS and MR strains as well as from D2S3 and MD females infected with DENV-2 JAM1409. The D2S3 and MD strains were infected with DENV-2 JAM1409 as described above. DENV infections and RNA extractions were performed in triplicate from each strain at 3 hr post-infection. Mid-guts were dissected and the blood meal removed as previously described from ∼10 infected individuals each for D2S3 and MD females and RNA was isolated using TRIzol Reagent (Invitrogen: http://www.invitrogen.com/). Control RNA was isolated from a pool of ∼30 mid-guts each from the uninfected blood fed MD and D2S3 females. First strand cDNA synthesis was performed using Superscript II Reverse Transcriptase (Invitrogen) according to manufacturer's instructions.

Primer Express Software version 3.0 (Applied Biosystems, Foster City, CA) was used to design primers. All amplifications and fluorescence quantification were performed using an ABI 7500 Fast System Sequence Detector System (Applied Biosystems) and the Sequence Detector Software version 1.3 (Applied Biosystems, Foster City, CA). The reactions were performed in a total volume of 25 µl containing 12.5 µl of SYBR Green PCR Master Mix, 10 ng of template, 300 nmol of each primer, and nuclease free water. Reactions were performed with the following conditions: 50°C for 2 min, 95°C for 10 min followed by 40 cycles of denaturation at 95°C for 15 s, annealing and extension at 60°C for 1 min. PCR efficiency was determined by amplifying serially diluted cDNA with each primer pair using the identical conditions. The log values of the template concentration versus the threshold cycle (*C*_T_) were used to plot the growth curve for the amplified products corresponding to each dilution. The slope of the curve was determined to quantify the efficiency of PCR. Efficiency greater than 0.95 was ensured for each qRT-PCR. The *C*_T_ value of each test gene relative to the reference gene, ribosomal protein S17 (*RpS17*), was used to determine the delta *C*_T_ values of infected sample and uninfected control. The *RpS17* gene was chosen as the reference gene based on previous results [24] and because it showed no changes in expression in our microarray data. Relative expression values were obtained using the delta-delta cycle threshold (ΔΔC_T_) method [25]. The *P*-values for testing differences in ΔC_T_ values between susceptible and refractory strains were derived using the nonparametric Wilcoxon two group test [26]. The null hypothesis assumed that ΔΔC_T_ was equal to 0, *P*-values <0.05 were considered significant.

## Results

### Identification of mosquito responsive genes to DENV infection

The *A. aegypti* genes responsive to the critical early stages of DENV infection were identified by a genome-wide transcriptome assay carried out using a NimbleGen oligonucleotide microarray format in MOYO-S (MS, susceptible to DENV) and MOYO-R (MR, refractory to DENV) females, upon challenging them with the JAM1409 strain of DENV (serotype-2). We chose to analyze gene expression at 3 hr and 18 hr post-exposure to DENV as the eventual susceptibility status of individual mosquitoes is likely defined during the first 24 hr. That is, DENV is known to rapidly enter vertebrate and insect cells via endocytic pathways [27], [28] and *in vivo* studies have reported that successful infection of midgut epithelial cells was already evident in ∼30% of midguts from three susceptible *A. aegypti* strains (including D2S3) by two days post-infection and thereafter spread laterally to infect neighboring cells [29].

The DENV-specific transcription response genes were identified by comparing each of the test samples (MS at 3 hr, MS at 18 hr, MR at 3 hr and MR at 18 hr) with the pooled uninfected control. An initial set of 6,339 genes were identified by Statistical Analysis of Microarray (SAM) [16] with significant (δ = 1.61, fdr = 0.52%) differential expression between the infected samples and uninfected control. Because we used a pooled reference microarray design, some of these differentially expressed genes were undoubtedly not related to DENV infection but instead were likely to be associated with developmental changes of the mosquitoes between 3 hr and 18 hr and/or differences between MS and MR strains related to feeding behavior, aging and other factors. To identify differentially expressed genes that were specifically responsive to *Ae. aegypt*-DENV interaction irrespective of time or strain differences, we selected for differentially expressed genes that were either up-regulated in both strains and time points or were down-regulated in both strains and time points in comparison to the common pool control. Using this pooled reference strategy, a total of 2,454 DENV responsive genes were identified from the initial set of 6,339 genes. Among the various commonly used strategies for microarray design [30], we felt that the pooled reference approach offered the most efficient and biologically relevant approach to uncovering only those genes directly associated with DENV infection.

Based on the observed expression levels of these significant genes, eight groups [(2 strain ×2 time points ×2 patterns of expression (up-regulation or down-regulation)] of non-overlapping genes were identified that constituted the DENV-specific transcription response genes (Table 1). All the genes within the individual groups showed differential expression levels corresponding to strain and time point. The levels of expression, however, varied from gene to gene as described in more detail below.

**Table 1:** Number of *Aedes aegypti* genes showing differential expression.

Strain	Time*	No. of genes	Expression
Moyo-S	3 hr	236	up-regulated
Moyo-S	3 hr	84	down-regulated
Moyo-R	3 hr	233	up-regulated
Moyo-R	3 hr	40	down-regulated
Moyo-S	18 hr	680	up-regulated
Moyo-S	18 hr	49	down-regulated
Moyo-R	18 hr	1021	up-regulated
Moyo-R	18 hr	111	down-regulated

*post infection with DENV.

### Modular networking patterns of host response genes

**Figure 1:**
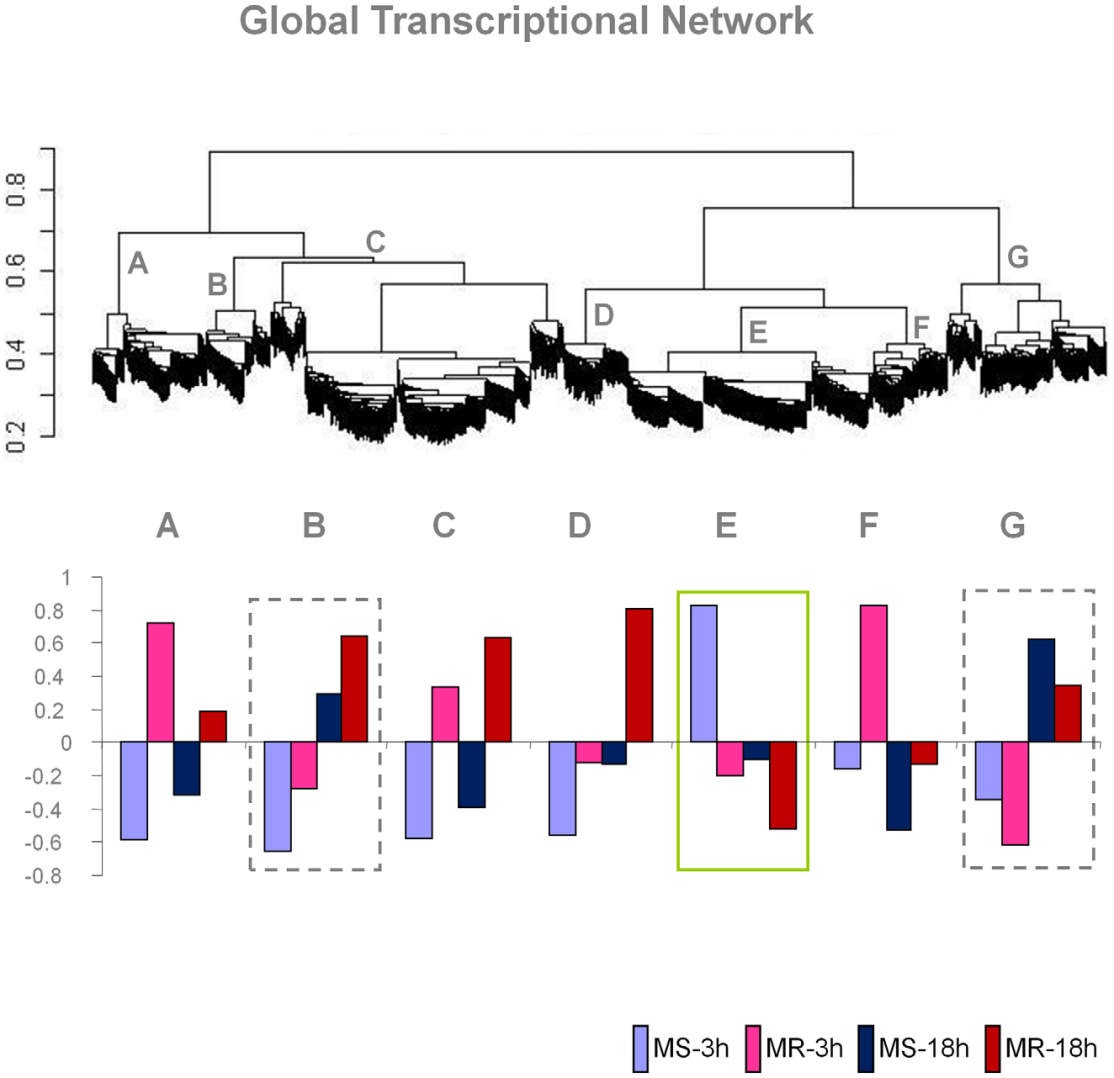
Modular expression patterns of *Aedes aegypti* genes in response to dengue virus infection. The top panel shows a WGCNA transcriptional network of host responsive genes between the susceptible (MS) and the refractory (MR) strains at 3 hr and 18 hr post-infection times, followed by dynamic hybrid cutting by topology overlapping. The scale on the left shows the branch heights. The modules are shown as ‘A’ through ‘G’. The bar graphs in the bottom panel represent the overall gene expression pattern of genes in the respective modules among the test samples. The up-regulated and down-regulated expression of genes is shown by eigengene values (module eigengene is defined as the first principal component of the expression matrix of the corresponding module) (shown in the Y-axis) for each module.

**Figure 2:**
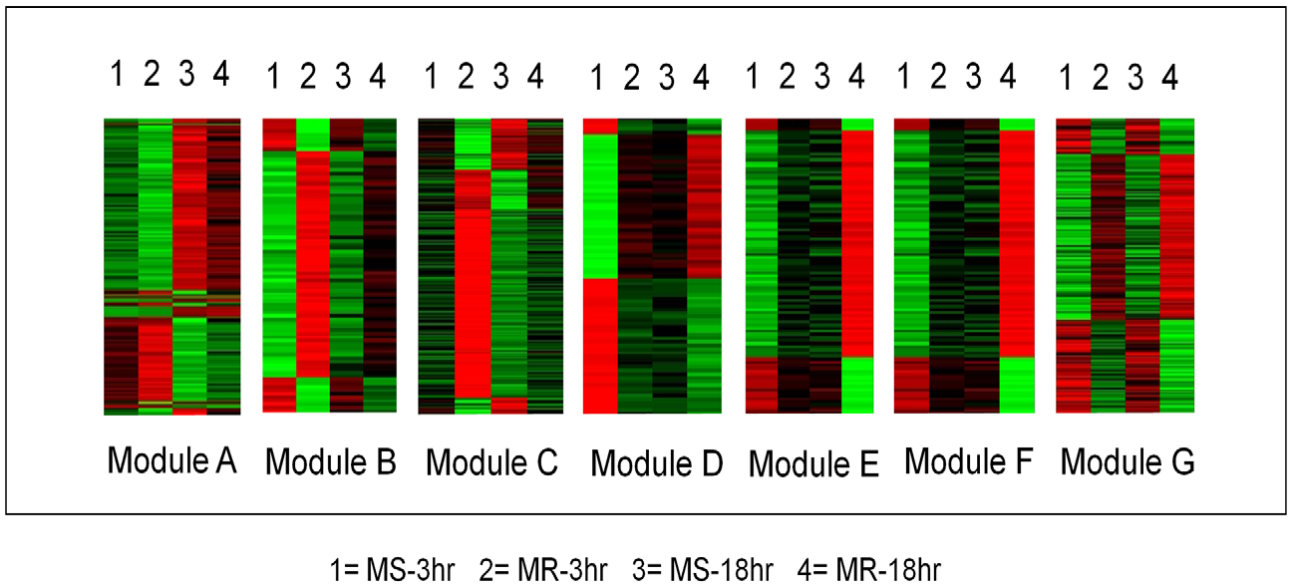
Heat maps of gene expression in the modules. Each row represents a gene in the module. The module IDs are indicated. The columns in the heat maps are labeled to indicate the mosquito strain and the time point to which the gene expression belongs to. The red color indicates up-regulation and green indicates down-regulation of gene expression.

Hierarchical clustering based on weighted gene co-expression network analysis (WGCNA) [19] identified extensive modular network patterns of *Ae. aegypti* genes in response to DENV infection. It was found that a total of 1,331 genes of the 2,454 responsive genes (54.2%) were involved in this global network, but in a modular manner. A total of seven ‘modules’ (designated as ‘A’ through ‘G’) of gene expression were predicted using a ‘dynamic hybrid cutting’ method [20] (Figure 1). A given gene in this network made interactions with as many as 52 other responsive genes as evident from the pair-wise gene interactions. The pair-wise gene interactions within and between these modules were predicted based on partial correlations (pcor) of gene expression by an empirical Bayes approach [21] using the GeneNet program (Table S1). The genes interacting within a module showed elevated average partial correlations among each other compared to that of genes interacting between modules (data not shown). Each module represented a group of genes with correlated expression patterns (Figure 2). For example, genes belonging to modules B and C have significantly similar expression patterns among the samples as compared to the genes belonging to modules D, E and F. Thus, B and C modules are clustered within one branch of the network cluster tree whereas modules D, E and F are clustered within the other branch of the tree (Figure 1). Module A and G, on the other hand, represent genes whose expression variations are quite distinct and hence are localized at the distal ends of the cluster tree.

**Figure 3:**
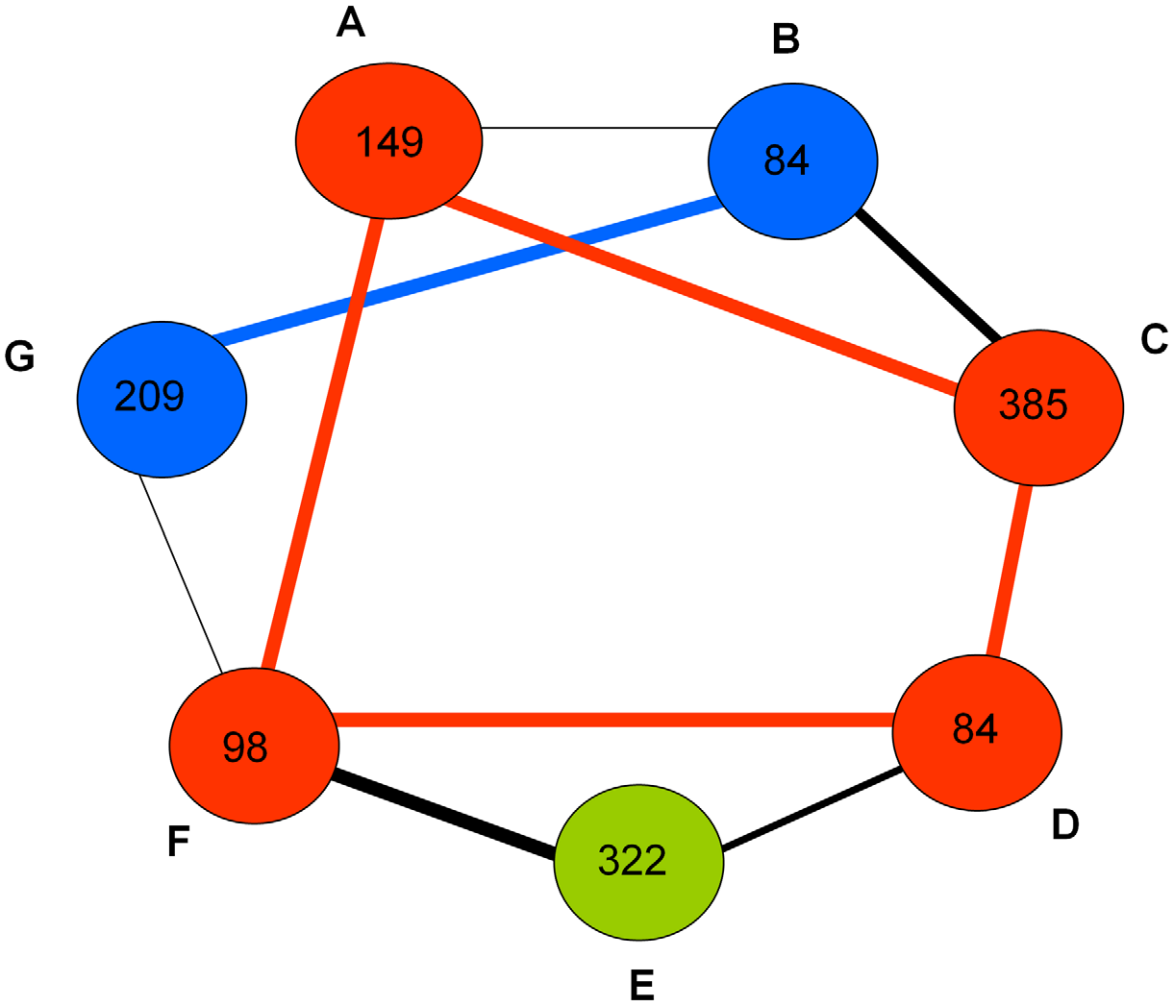
A simplified illustration of inter-modular networking of *Aedes aegypti* genes responsive genes. The expression modules are shown by colored circles (A through G). The number of genes belonging to each module is shown. The black lines connect the modules. The thickness of these line shows how closely similar they are to each other in the cluster tree (see Figure 1). The red colored circles and lines represent the refractory response modules (RRM), the green color circle represents the susceptible response module (SRM) and the blue colored circles and lines represent the core response modules (CRM).

The observed patterns of pair-wise interactions within and between the predicted expression modules showed that about two-thirds of all the genes in the network interacted in an inter-modular manner indicating extensive cross-talk among the modules. The numbers of interacting genes that formed the connectivity among these modules were highly variable (Figure 3). The genes that responded coordinately to DENV infection in the susceptible MS strain were predominantly clustered in one specific expression module (module E; susceptible response module or SRM), whereas in the refractory MR strain they were distributed in modules A, C, D and F (refractory response module or RRM) (Figure 1). Modules B and G represent genes that show time specific differential expression (between 3 hr and 18 hr post infection) in both the susceptible and refractory strains (see Figure 2). These genes may be involved in triggering a common host response (core response module, CRM) in the early stages of infection in both strains. A list of responsive genes specific to individual predicted modules is provided in Table S2.

### Enrichment of pathway genes within expression modules

In order to analyze the functional attributes of these expression modules, we made use of *A. aegypti* KEGG pathways to determine if the modularly expressed genes represented specific pathway(s). Of all the KEGG pathways that were mapped to each expression module, genes of specific pathway(s) were predominant within the predicted expression modules (Table 2). All the predicted pathways associated with the seven expression modules (except module E where p<0.1) were significantly (p<0.05) enriched with genes representing specific pathways.

**Table 2:** Representation of pathway genes in expression modules.

Expression Module	KEGG pathway genes representing the expression module*	p-value
A	Carbohydrate Metabolism (30 /83)	0.029
B	Glycan Biosynthesis/Metabolism (30 /78)	0.015
C	Lipid Metabolism (45 /114)	0.003
	Metabolism of Cofactors /Vitamins (34 /98)	0.035
	Nucleotide Metabolism (47 /119)	0.002
D	Xenobiotics Biodegradation /Metabolism (31 /80)	0.013
E	Energy Metabolism (23 /83)	0.098
	Replication /Repair (21 /79)	0.095
	Biosynthesis of Secondary Metabolites (21 /81)	0.089
F	Amino Acid Metabolism (36 /77)	0
	Cell Growth/ Death (25 /63)	0.016
G	Signal Transduction (76 /174)	0
	Sorting /Degradation (22 /56)	0.024

*The numbers in the parenthesis show the counts of responsive genes identified from the expression network analysis versus the total number of genes annotated to the specific category of KEGG pathways.

The susceptible response module (module E) showed enrichment with genes related to energy metabolism and DNA replication and repair. The over-representation of genes of the DNA replication pathway in the susceptible mosquitoes may be related to activities associated with the DENV infection process. Indeed, it has been shown that the cell cycle environment in C6/36 cells influences the course of DENV infection wherein DENV replication is enhanced in S-phase cells [31], and blood feeding alone activates cellular metabolism and is known to induce S-phase in multiple tissues in adult females [32],[33].

The refractory response modules (A, C, D and F) showed significant enrichment with different metabolism pathway genes that included cytochrome P450 genes, genes involved in DDT [1,1,1-Trichloro-2,2-bis(4-chlorophenyl) ethane] degradation (mostly including the short-chain dehydrogenase, amino acid decarboxylase as well as glutathione-s-transferase theta (gst) coding genes) and also genes associated with cell growth and death, such as cell division and apoptosis. Several p53 signaling genes, caspase genes and phosphatidylinositol 3-kinase signaling genes were up-regulated in the refractory response modules (Table 3). These pathways are known to modulate apoptosis in response to viral infections from other studies. Because such evidence is presently restricted to vertebrate cells [34]–[39], further studies are needed to confirm their roles in insects.

**Table 3:** List of p53 and apoptosis related genes.

Gene	Description	Expression*
AAEL009572	cyclin B3	MR (u); 3.7; 1.3
AAEL006300	P53 induced protein	MR (u); 3.4; 1.4
AAEL010967	tetraspanin	MR (u); 3.2; 1.5
AAEL014311	tetraspanin	MR (u); 4; 1.5
AAEL002487	P53 regulated pa26 nuclear protein sestrin	MR (u); 3.4; 1.3
AAEL005646	vitellogenin, putative	MR (u); 6.5; 1.4
AAEL007642	interleukin-1 receptor-associated kinase 4	MR (u); 3.4; 1.3
AAEL008823	RAC serine/threonine-protein kinase	MR (u); 7.2; 1.3
AAEL002277	cAMP-dependent protein kinase regulator	MR (u); 5.7; 1.5
AAEL008930	protein phosphatase 3, catalytic subunit	MR (u); 6.3; 1.4
AAEL012143	caspase1	MR (u); 3.1; 2.2
AAEL005963	caspase3	MR (u); 3.1; 1.7
AAEL004379	phosphatidylinositol 4-kinase	MR (u); 7.1; 1.3
AAEL009294	phosphatidylinositol 4-kinase	MR (u); 5.4; 1.6
AAEL015255	1-phosphatidylinositol-4-phosphate 5-kinase	MR (u); 4.2; 1.3
AAEL008510	diacylglycerol kinase	MR (u); 4.8; 2.1
AAEL004285	diacylglycerol kinase	MR (u); 5; 2.1
AAEL013183	1D-myo-inositol-triphosphate 3-kinase	MR (u); 5.5; 1.3
AAEL000386	phosphatidylinositol-4-phosphate 3-kinase	MR (u); 8; 1.3
AAEL001940	phosphatidate cytidylyltransferase	MR (u); 3.3; 1.3
AAEL003143	phosphatidylinositol-bisphosphatase	MR (u); 5.6; 1.2
AAEL011417	inositol-1,4,5-trisphosphate 5-phosphatase	MR (u); 11.3; 1.5
AAEL013891	inositol-1,4,5-trisphosphate 5-phosphatase	MR (u); 5.3; 1.4
AAEL014247	inositol-1,4,5-trisphosphate 5-phosphatase	MR (u); 5.5; 1.5
AAEL014358	inositol polyphosphate-4-phosphatase	MR (u); 2.6; 2
AAEL001108	classical protein kinase C	MR (u); 8.8; 2

*The letter ‘u’ or ‘d’ in the bracket shows if the gene is up-regulated or down-regulated in the infected mosquitoes with respect to uninfected control. The two numbers after that represent the SAM score and the fold-change of expression of the gene from the array expression data.

**Figure 4:**
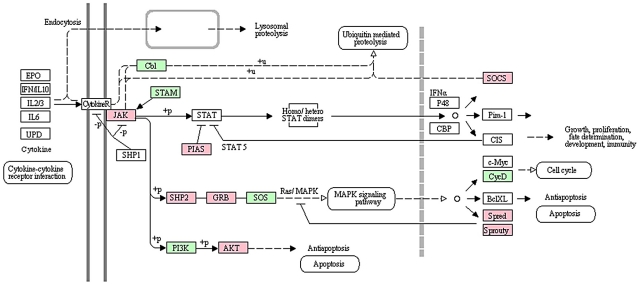
Relative abundance of different signal transducing genes responsive to dengue infection in *Aedes aegypti*. The percentage below each category represents the percentage of genes of that category with respect to all responsive genes related to signal transduction (based on KEGG pathways).

**Figure 5:**
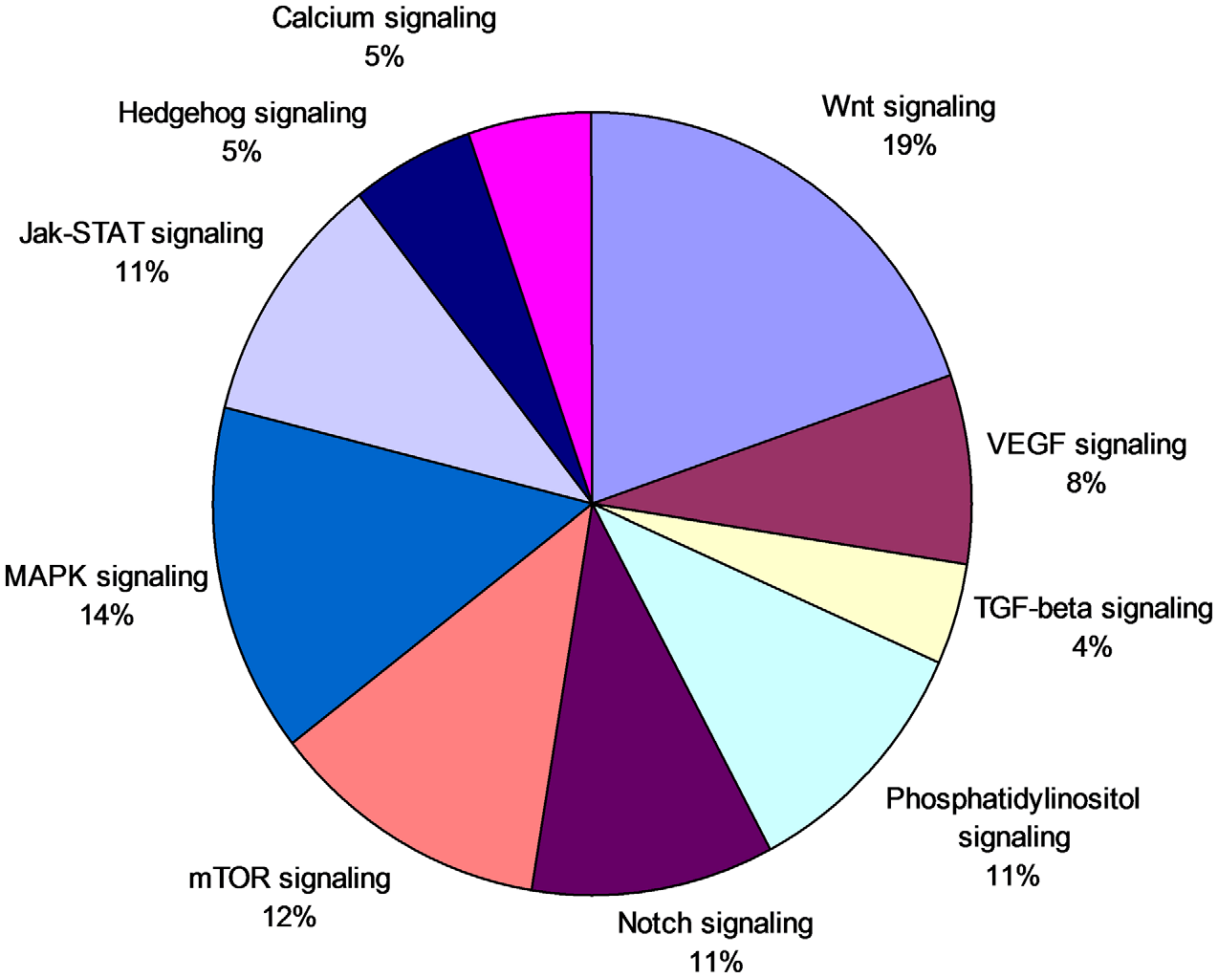
A pathway diagram of JAK-STAT cascades of dengue responsive genes. The KEGG pathway template was used to generate this diagram by mapping the responsive genes (genes responsive in MS strain are in pink and genes responsive in MR strain are in green).

The common core response modules (B and G) were enriched with genes related to signal transduction, as well as sorting and degradation. The mechanisms of how the gene networks transduce signals to trigger the appropriate host action in *A. aegypti* against DENV infection are important aspects of vector competence. Genes related to important signal transduction pathways such as the Wnt, MAPK, mTOR and JAK-STAT pathways were predominant among all the responsive signal transduction pathway genes (Figure 4). We identified several genes associated with the JAK-STAT pathway among the responsive genes (Table 4). Significant differential expression of *A. aegypti* genes of this pathway may be involved in the activation of the STAT in response to induction by JAK (Figure 5). In addition to the activation of STAT, JAK induction may mediate the recruitment of other molecules such as the MAP kinases which results in the activation of additional transcription factors. It is possible that the JAK-STAT signaling pathway may be involved in activating the MAPK cascade [40]–[41] or in regulating apoptosis as shown in *Drosophila* [42]. The interface of these core response genes with genes involved in susceptible response and refractory response module(s) suggests their important roles in the global-cross talk among the host factors during these early infection periods that could trigger the appropriate host action in the susceptible and refractory mosquitoes.

**Table 4 pntd-0001385-t004:** List of JAK-STAT pathway genes.

Gene	Description	Expression*
AAEL012553	Janus kinase	MS (u); 5.7; 1.9
AAEL000393	suppressors of cytokine signaling	MS (u); 3.7; 1.7
AAEL008528	protein tyrosine phosphatase n11 (shp2)	MS (u); 2.9; 1.8
AAEL003532	sprouty-related, EVH1 domain containing	MS (u); 4; 1.5
AAEL014510	sprouty	MS (u); 3.4; 1.7
AAEL015099	sumo ligase	MS (u); 6; 2.1
AAEL009692	signal transducer and activator of transcription	MR (u); 9.2; 1.5
AAEL006936	suppressor of cytokine signaling, invertebrate	MR (u); 3.1; 1.3
AAEL013786	growth factor receptor-bound protein	MR (u); 4; 1.3
AAEL014394	growth factor receptor-bound protein	MR (u); 4.1; 1.3
AAEL008823	rac serine/threonine kinase	MR (u); 7.2; 1.3

*The letter ‘u’ or ‘d’ in the bracket shows if the gene is up-regulated or down-regulated in the infected mosquitoes with respect to uninfected control. The two numbers after that represent the SAM score and the fold-change of expression of the gene from the array expression data.

Vacuolar trafficking upon DENV entry into a mosquito mid-gut epithelial cell may be an important cellular process associated with mosquito-virus interaction. Such mechanisms have been described wherein pH-dependent vacuolar trafficking modulates flaviviral entry into human as well as mosquito cells [43]–[46]. Consistent with this likelihood, our data suggests the potential for differential expression of the endocytic pathway between susceptible and refractory strain in response to dengue infection. We observed that genes coding for V-ATPases are differentially expressed between the susceptible and refractory strain. That is, of the 17 differentially expressed V-ATPases genes, 15 genes were up-regulated in the susceptible strain (Table 5). It is plausible that differential expression of these genes could influence the endocytic pathway, possibly by differential acidification of endosomes [44].

**Table 5 pntd-0001385-t005:** List of V-ATPase genes.

Gene	Description	Expression*
AAEL000291	vacuolar ATPases	MS (u); 2.7; 1.3
AAEL008787	vacuolar ATPases	MS (u); 5.3; 1.7
AAEL012113	vacuolar ATPases	MS (u); 2.3; 1.2
AAEL011025	vacuolar ATPases	MS (u); 3.4; 1.7
AAEL005798	vacuolar ATPases	MS (u); 4.4; 1.6
AAEL015594	vacuolar ATPases	MS (u); 2.9; 1.3
AAEL012035	vacuolar ATPases	MS (u); 3.9; 1.7
AAEL013302	vacuolar ATPases	MS (u); 2.9; 1.3
AAEL007184	vacuolar ATPases	MS (u); 2.9; 1.3
AAEL012819	vacuolar ATPases	MS (u); 3.6; 1.6
AAEL006516	vacuolar ATPases	MS (u); 3.6; 1.6
AAEL010819	vacuolar ATPases	MS (u); 3.0; 1.3
AAEL014053	vacuolar ATPases	MR (u); 4.2; 1.2
AAEL003743	vacuolar ATPases	MR (u); 3; 1.2

*The letter ‘u’ or ‘d’ in the bracket shows if the gene is up-regulated or down-regulated in the infected mosquitoes with respect to uninfected control. The two numbers after that represent the SAM score and the fold-change of expression of the gene from the array expression data.

Our data also revealed that cuticle protein (CP) genes may play important roles in *A. aegypti* response to dengue infection. We observed that while a total of 28 responsive cuticle protein (CP) genes were down-regulated among the strains upon DENV infection (Table 6), the extent of down-regulation of these genes was much more severe in the MS strain (n = 25 genes) than the MR strain (n = 3genes); thus these genes showed proportionally higher overall expression levels in MR mosquitoes than the MS mosquitoes. Although the function of cuticle protein in dengue infection is not known, it is possible that they may play a role in development of anatomical barriers for virus dissemination as an additional innate defense mechanism in the refractory mosquitoes.

**Table 6:** List of cuticle protein genes.

Gene	Description	Expression*
AAEL014416	pupal cuticle protein 78E	MR (d); −5.8; 0.7
AAEL008988	adult cuticle protein	MS (d); −2.9; 0.8
AAEL015223	adult cuticle protein	MR (d); −3.4; 0.5
AAEL008999	adult cuticle protein	MR (d); −4.9; 0.6
AAEL000665	adult cuticle protein	MS (d); −4.2; 0.7
AAEL008983	adult cuticle protein	MS (d); −3.8; 0.8
AAEL014982	adult cuticle protein	MS (d); −3.8; 0.8
AAEL009790	cuticle protein	MS (d); −3.8; 0.6
AAEL009791	cuticle protein	MS (d); −3.6; 0.7
AAEL015364	cuticle protein	MS (d); −4; 0.7
AAEL015363	cuticle protein	MS (d); −3; 0.7
AAEL000878	cuticle protein	MS (d); −3.5; 0.7
AAEL009802	cuticle protein	MS (d); −2.9; 0.7
AAEL007101	adult cuticle protein	MS (d); −5.1; 0.5
AAEL009793	cuticle protein	MS (d); −5.1; 0.5
AAEL000879	cuticle protein	MS (d); −4.9; 0.6
AAEL009800	cuticle protein	MS (d); −4.8; 0.7
AAEL008295	pupal cuticle protein 78E	MS (d); −3.2; 0.7
AAEL008284	pupal cuticle protein 78E	MS (d); −3.2; 0.7
AAEL008288	pupal cuticle protein 78E	MS (d); −5.1; 0.5
AAEL004751	pupal cuticle protein	MS (d); −5.8; 0.6
AAEL013512	pupal cuticle protein	MS (d); −5.8; 0.6
AAEL004780	pupal cuticle protein	MS (d); −5.7; 0.7
AAEL004781	pupal cuticle protein	MS (d); −5.2; 0.4
AAEL004771	pupal cuticle protein	MS (d); −4.9; 0.6
AAEL004747	pupal cuticle protein	MS (d); −4.9; 0.6
AAEL013520	pupal cuticle protein	MS (d); −2.9; 0.8
AAEL007192	pupal cuticle protein	MS (d); −3.1; 0.8

*The letter ‘u’ or ‘d’ in the bracket shows if the gene is up-regulated or down-regulated in the infected mosquitoes with respect to uninfected control. The two numbers after that represent the SAM score and the fold-change of expression of the gene from the array expression data.

### Validation of gene expression by qRT-PCR

**Figure 6:**
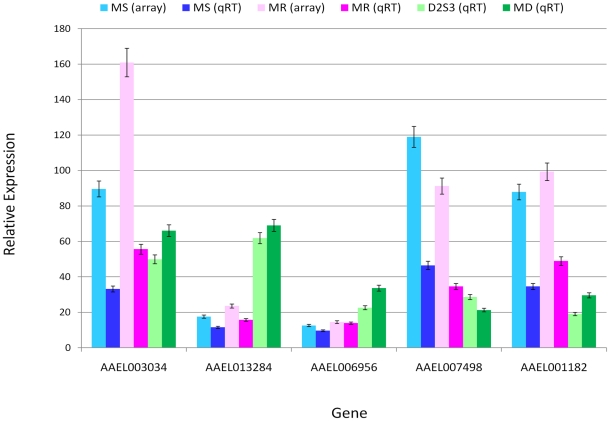
Validation of microarray expression of five genes by quantitative real-time PCR (qRT-PCR). For each gene, comparisons are made between array data (MS and MR strains) and the qRT-PCR data (MS and MR strains). Also, two additional strains of *A. aegypti*, D2S3 and MD, were infected with dengue (see text) and then compared for expression of the same genes by qRT-PCR. The relative gene expression (in comparison to the control) is shown in the Y-axis along with standard errors. The X-axis shows the gene identity.

To validate the microarray data and to determine if genes differentially expressed between the MS and MR strains in response to DENV infection are also involved in driving similar transcriptional responses in other *A. aegypti* strains upon DENV infection, we performed quantitative real time PCR (qRT-PCR) assays. Five genes were randomly chosen from the microarray data set (Table S3) and subjected to qRT-PCR in the MS and MR strains, and two additional *A. aegypti* strains (D2S3: DENV susceptible and Moyo-in-Dry or MD: DENV refractory). After challenge with DENV-2 JAM1409, D2S3 and MD samples at 3 hr post-infection were quantified by qRT-PCR for all five genes and compared with results for both microarray and qRT-PCR with the MS and MR strains (Figure 6). The qRT-PCR data showed statistically significant (P<0.05) up-regulated or down-regulated expression patterns for each gene between the MS and MR strains as well as between the D2S3 and MD strains with respect to the uninfected control. Comparisons of the microarray and qRT-PCR results showed consistent trends in variation (R^2^>0.9 and P<0.05; Figure S1). These observations indicate that the DENV responsive genes may have similar susceptibility-specific host responses to DENV infection in different *A. aegypti* strains and may play important roles in vector competence to DENV infection at the critical early infection stages.

## Discussion

We conducted a comparative genome-wide survey of gene expression patterns observed in response to DENV infection among *A. aegypti* females known to be susceptible or refractory to infection. Our results show that 2,454 DENV responsive genes interact in well-defined patterns that distinguish the two response phenotypes. The observed transcriptional network establishes global cross-talk among the DENV response genes that may subsequently trigger the appropriate host actions at the critical early time points following exposure to and infection by DENV.

Our data revealed that 293 genes were responsive in both susceptible and refractory strains, whereas most DENV responsive genes had expression patterns that were specific to either susceptible or refractory genotypes (Figure 3). It also showed that the refractory phenotype involved a much greater number of genes that acted in tightly correlated manners relative to the number of genes which were associated with the susceptible phenotype. This indicated that *A. aegypti* may utilize more complex defense mechanisms compared to those required to host the virus. This is also clearly seen from data shown in Table 1. Based on the correlated expression levels of the responsive genes, we identified several candidate pathways that may determine the compatible or non-compatible interaction between *A. aegypti* and DENV. Most of these pathway genes were associated with expression fold-changes ranging from 0.5 to 2.2 (Tables 3–6) indicating that some important pathways show relatively modest transcriptional responses to DENV infection. We did, however, observe other DENV responsive genes with much higher transcriptional responses (Table S4).

The length of the extrinsic incubation (EIP, total time from viral entry to transmission stage) period likely varies depending upon the host and viral genotype, environmental factors such as temperature and humidity, as well as other unknown intrinsic factors [47]. Recent evidence demonstrates that, depending on genetic background of the *A. aegypti* population, the EIP can be completed in as few as 4 days in contrast to 7 to 10 days as most commonly observed [29].

As observed in a recent study that examined transcriptome response to DENV infection in a susceptible *A. aegypti* strain at seven days post-infection [48], the involvement of the JAK-STAT pathway in controlling virus infection was evident in our study. Another recent study identified candidate genes in a susceptible *A. aegypti* strain at 10 days post-infection [49], and determined that the Toll and the JAK/STAT pathways play important roles in controlling DENV infection in *A. aegypti*. In *Drosophila*, the JAK-STAT pathway has been shown to be necessary but not sufficient for triggering an anti-viral defense in *Drosophila* to Drosophila C virus (DCV) infection [50]. Our data, that focused on the critical early time points for infection and compared DENV susceptible and refractory strains, revealed that genes of the JAK-STAT pathway were up-regulated in response to DENV challenge in both the MS and MR strains at these early periods, suggesting that while it does play a role in determining DENV infection its significance in defining vector competence remains unclear.

We observed activation of JNK and p53 related genes as well as caspase genes in response to DENV infection in the MR strain. This suggests possible induction of programmed cell death events in the refractory strain following DENV infection. Programmed cell death is an efficient host survival mechanism in insects where the infected cells undergo apoptosis to prevent viral infections [51]. Moreover, a role for apoptosis has been implicated in mosquito response to infection by several arboviruses and orthologs to apoptosis-associated genes in *Drosophila* have been identified and are expressed in *A. aegypti* [52]. Although a caspase-dependent role in apoptosis induction has been suggested in dengue virus infection in animal cells [36], [38], [53], further studies are however required to determine if programmed cell death is one of the mechanisms of controlling DENV infection in *A. aegypti*.

We also identified several DENV responsive genes that were previously reported to play important roles in modulating viral infections in animal cells. For example, a trypsin gene (AAEL010195) was significantly down-regulated gene in both strains (data not shown). In *A. aegypti*, midgut trypsins have been shown to influence the rate of DENV-2 infection and dissemination [54]. In addition, another serine protease (AAEL005753) was similarly significantly down-regulated in the infected mosquitoes of both strains. Serine proteases play an important role in proteolytic digestion of blood meal proteins in mosquitoes, and results have shown that some midgut serine proteases may play a role in DENV-2 infectivity of *A. aegypti* [55]. We also identified a Toll-like receptor gene (AAEL015018) as significantly up-regulated in both strains. Toll-like receptors are well known genes that have been shown to invoke anti-viral innate immune responses or to ameliorate viral infection in various host cells including *A. aegypti* in response to DENV [49], [56]. Additionally, two furin-like genes (AAEL010725 and AAEL002317) were also found over-expressed in the MS strain only that may be involved in efficient maturation of newly synthesized virions by cleavage of the DENV precursor membrane protein, prM [57].

We observed that while cuticle protein genes were significantly down-regulated following DENV infection in both the MS and MR strains, the majority (25 of 28 genes) were down-regulated in the MS strain. It is plausible that the generally higher expression levels of these genes in the MR strain may be associated with enhanced anatomical barriers in these mosquitoes, possibly by the tracheal system, to limit DENV escape from the mid-gut epithelium. In this regard, it is interesting to note that the reported anatomical barriers to DENV transmission in *A. aegypti* include a mid-gut escape barrier (MEB), wherein the virus may be able to successfully invade and replicate in the mid-gut epithelium but is blocked from disseminating to other tissues [47]. The tracheal system makes intimate contact with mid-gut epithelial cells and has been identified as a dissemination conduit for several insect/virus systems, including *A. aegypti* and DENV [29]. Tracheae do contain a cuticular lining that could limit virus dissemination [58] and, therefore, dissemination from the mid-gut could be impacted by differential induction of cuticle proteins in the MR strain.

Our data suggest, based on numbers of genes and the diversity of metabolic pathways involved, that defending against viral infection reflects greater evolutionary complexity. The resilient nature of *A. aegypti* as the primary vector for dengue transmission is apparent [1] and thus, understanding gene expression patterns across various natural populations is needed to provide insights on genome-wide networking of DENV responsive genes, and the ultimate impact on population-specific vector competence. It is expected that comparative analyses at the population level may identify genes within differential network patterns critical to a susceptible or refractory response. Such variation may uncover key points in metabolic pathways for development of novel intervention strategies. Future efforts need to be directed toward better clarification of the specific roles of individual pathways and well as identification of key points for their interactions using systems biology approaches [59].

## Supporting Information

Figure S1 Highly similar expression pattern between microarray and qRT-PCR results.
(TIF)

Table S1 An example of pair-wise gene interactions predicted by GeneNet software.
(DOCX)

Table S2 List of selected genes that are expressed in modular manner. The associations of these genes to KEGG pathways are shown.
(DOCX)

Table S3 List of genes and primer sequences (5′-3′) used for qRT-PCR validations.
(DOCX)

Table S4 List of significant DENV responsive genes that showed higher fold-change than the majority of genes belonging to the predicted modules wherein correlated expression patterns were observed.
(DOCX)
