# Supplementary figures and images for: Global Cross-Talk of Genes of the Mosquito Aedes aegypti in Response to Dengue Virus Infection
Source: PLoS Negl Trop Dis. 2011 Nov 15;5(11):e1385. doi: 10.1371/journal.pntd.0001385 (PMC3216916; doi:10.1371/journal.pntd.0001385)

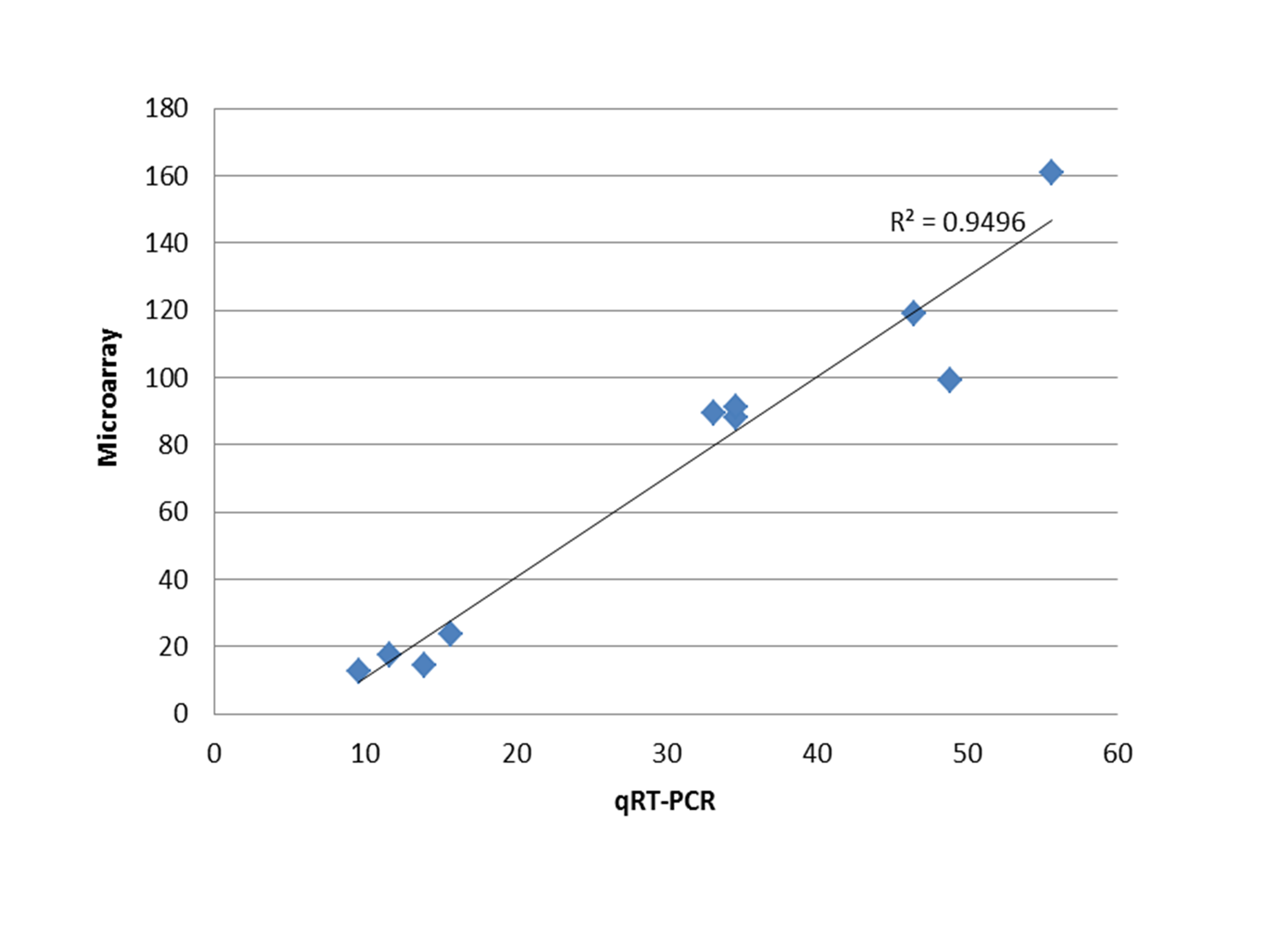

Supplement: Figure S1 — Highly similar expression pattern between microarray and qRT-PCR results. (TIF) [file pntd.0001385.s001.tif]
