# Supplementary material for: Global Cross-Talk of Genes of the Mosquito Aedes aegypti in Response to Dengue Virus Infection
Source: PLoS Negl Trop Dis. 2011 Nov 15;5(11):e1385. doi: 10.1371/journal.pntd.0001385 (PMC3216916; doi:10.1371/journal.pntd.0001385)
Supplement: Table S1 — An example of pair-wise gene interactions predicted by GeneNet software. (DOCX) [file pntd.0001385.s002.docx]

Table S1. Example of pair-wise gene interactions predicted by GeneNet software. The interacting gene pairs were extracted from the .dot output file. Only a subset of the responsive genes is shown here to present as an example. The gene shown in column 1 (Gene1) interacts with one or more genes (Gene2) shown in the second column, some of these interactions being strong and others being weak as well.

| **Gene1** | **Gene2** | **Interaction** |
| --- | --- | --- |
| AAEL000242 | AAEL013900 | Strong |
|  | AAEL012106 | Strong |
|  | AAEL002127 | Strong |
|  | AAEL008816 | Strong |
|  | AAEL015639 | Weak |
|  | AAEL006054 | Strong |
|  | AAEL010195 | Weak |
| AAEL000381 | AAEL013763 | Strong |
|  | AAEL013489 | Strong |
| AAEL001467 | AAEL010881 | Strong |
| AAEL002127 | AAEL012106 | Strong |
|  | AAEL013900 | Strong |
|  | AAEL008055 | Strong |
|  | AAEL008816 | Strong |
|  | AAEL010783 | Strong |
| AAEL002138 | AAEL014021 | Strong |
|  | AAEL014792 | Strong |
| AAEL002535 | AAEL015639 | Weak |
| AAEL002724 | AAEL015639 | Strong |
|  | AAEL008010 | Strong |
|  | AAEL013763 | Strong |
| AAEL005493 | AAEL007245 | Strong |
| AAEL006054 | AAEL013900 | Strong |
|  | AAEL014792 | Strong |
| AAEL006966 | AAEL010783 | Strong |
|  | AAEL007245 | Weak |
| AAEL007245 | AAEL010783 | Weak |
| AAEL008010 | AAEL013763 | Strong |
|  | AAEL015639 | Strong |
|  | AAEL008695 | Strong |
| AAEL008055 | AAEL013900 | Strong |
|  | AAEL008816 | Strong |
|  | AAEL012106 | Strong |
|  | AAEL008695 | Strong |
| AAEL008695 | AAEL015639 | Strong |
|  | AAEL010881 | Strong |
| AAEL008816 | AAEL012106 | Strong |
|  | AAEL013900 | Strong |
| AAEL008925 | AAEL010694 | Strong |
| AAEL010195 | AAEL013900 | Weak |
| AAEL010783 | AAEL012106 | Strong |
|  | AAEL014792 | Weak |
|  | AAEL014591 | Strong |
| AAEL010881 | AAEL013763 | Strong |
| AAEL012106 | AAEL013900 | Strong |
| AAEL013763 | AAEL015639 | Strong |
| AAEL013900 | AAEL014792 | Strong |
| AAEL014021 | AAEL014591 | Strong |
|  | AAEL015125 | Strong |
| AAEL014792 | AAEL015262 | Strong |
