# Supplementary material for: Global Cross-Talk of Genes of the Mosquito Aedes aegypti in Response to Dengue Virus Infection
Source: PLoS Negl Trop Dis. 2011 Nov 15;5(11):e1385. doi: 10.1371/journal.pntd.0001385 (PMC3216916; doi:10.1371/journal.pntd.0001385)
Supplement: Table S2 — List of selected genes that are expressed in modular manner. The associations of these genes to KEGG pathways are shown. (DOCX) [file pntd.0001385.s003.docx]

Table S2. List of selected genes that are expressed in modular manner. The association of these genes to KEGG pathways are shown. The MM value (Module Membership value) and GS (Gene Significance) value represent the statistical parameters used by WGCNA program to assign genes to specific modules based on their expression. The listed genes were selected with MM > 0.432 and GS > 0.528 as they were centrally localized within each module. The genes of F module are not listed as the MM and GS values were smaller than the selection criteria (user defined).

| Module | Gene | Gene description | MM value | GS value | Association with KEGG pathway |
| --- | --- | --- | --- | --- | --- |
| A | AAEL007839 | beta-hexosaminidase | 0.997 | 0.950 | Carbohydrate Metabolism |
| A | AAEL002430 | n-acetylglucosamine-6-phosphate deacetylase | 0.964 | 0.839 | Carbohydrate Metabolism |
| A | AAEL011597 | hypothetical protein | 0.967 | 0.931 | Carbohydrate Metabolism |
| A | AAEL009782 | brain chitinase and chia | 0.935 | 0.697 | Carbohydrate Metabolism |
| A | AAEL003083 | myotubularin | 0.816 | 0.914 | Carbohydrate Metabolism |
| A | AAEL011597 | hypothetical protein | 0.967 | 0.931 | Carbohydrate Metabolism |
| A | AAEL001480 | phosphoglucomutase | 0.899 | 0.903 | Carbohydrate Metabolism |
| A | AAEL003083 | myotubularin | 0.816 | 0.914 | Carbohydrate Metabolism |
| A | AAEL010536 | alpha-amylase | 0.927 | 0.648 | Carbohydrate Metabolism |
| A | AAEL012489 | hypothetical protein | 0.954 | 0.948 | Carbohydrate Metabolism |
| A | AAEL000321 | acetyl-coa synthetase | 0.940 | 0.596 | Carbohydrate Metabolism |
| A | AAEL014707 | 2-hydroxyphytanoyl-coa lyase | 0.811 | 0.743 | Carbohydrate Metabolism |
| A | AAEL015255 | phosphatidylinositol-4-phosphate 5-kinase type i | 0.969 | 0.748 | Carbohydrate Metabolism |
| A | AAEL014358 | hypothetical protein | 0.943 | 0.617 | Carbohydrate Metabolism |
| A | AAEL014247 | type i inositol-1,4,5-trisphosphate 5-phosphatase | 0.954 | 0.748 | Carbohydrate Metabolism |
| A | AAEL009294 | phosphatidylinositol 4-kinase | 0.983 | 0.830 | Carbohydrate Metabolism |
| A | AAEL013891 | type i inositol-1,4,5-trisphosphate 5-phosphatase | 0.844 | 0.966 | Carbohydrate Metabolism |
| A | AAEL001586 | glucosyl/glucuronosyl transferases | 0.863 | 0.723 | Carbohydrate Metabolism |
| A | AAEL000321 | acetyl-coa synthetase | 0.940 | 0.596 | Carbohydrate Metabolism |
| A | AAEL000321 | acetyl-coa synthetase | 0.940 | 0.596 | Carbohydrate Metabolism |
| A | AAEL011744 | DEAD box ATP-dependent RNA helicase | 0.960 | 0.953 | Carbohydrate Metabolism |
| A | AAEL001586 | glucosyl/glucuronosyl transferases | 0.863 | 0.723 | Carbohydrate Metabolism |
| A | AAEL011187 | U520 | 0.969 | 0.637 | Carbohydrate Metabolism |
| A | AAEL010536 | alpha-amylase | 0.927 | 0.648 | Carbohydrate Metabolism |
| A | AAEL004676 | (s)-2-hydroxy-acid oxidase | 0.699 | 0.895 | Carbohydrate Metabolism |
| A | AAEL010505 | ribulose-5-phosphate-3-epimerase | 0.983 | 0.573 | Carbohydrate Metabolism |
| A | AAEL004002 | glucose dehydrogenase | 0.909 | 0.953 | Carbohydrate Metabolism |
| A | AAEL010505 | ribulose-5-phosphate-3-epimerase | 0.983 | 0.573 | Carbohydrate Metabolism |
| A | AAEL005744 | DEAD box ATP-dependent RNA helicase | 0.989 | 0.902 | Carbohydrate Metabolism |
| A | AAEL001478 | bile acid beta-glucosidase, putative | 0.917 | 0.918 | Carbohydrate Metabolism |
| B | AAEL008509 | alanyl-tRNA synthetase | 0.982 | 0.569 | Amino Acid Metabolism |
| B | AAEL002748 | aspartyl-tRNA synthetase | 0.971 | 0.758 | Amino Acid Metabolism |
| B | AAEL011355 | carnitine o-acetyltransferase | 0.926 | 0.551 | Amino Acid Metabolism |
| B | AAEL005801 | bifunctional aminoacyl-tRNA synthetase | 0.852 | 0.983 | Amino Acid Metabolism |
| B | AAEL008418 | pyrroline-5-carboxylate reductase | 0.950 | 0.833 | Amino Acid Metabolism |
| B | AAEL000716 | chondroitin 4-sulfotransferase | 0.988 | 0.983 | Amino Acid Metabolism |
| B | AAEL007480 | glutamate-cysteine ligase, regulatory-subunit, putative | 0.950 | 0.985 | Amino Acid Metabolism |
| B | AAEL009490 | carbamoyl-phosphate synthase large chain | 0.916 | 0.899 | Amino Acid Metabolism |
| B | AAEL010276 | aminomethyltransferase | 0.694 | 0.645 | Amino Acid Metabolism |
| B | AAEL012764 | hypothetical protein | 0.961 | 0.814 | Amino Acid Metabolism |
| B | AAEL014936 | sarcosine dehydrogenase | 0.779 | 0.964 | Amino Acid Metabolism |
| B | AAEL010807 | hypothetical protein | 1.000 | 0.689 | Amino Acid Metabolism |
| B | AAEL004290 | histone-lysine n-methyltransferase | 0.896 | 0.754 | Amino Acid Metabolism |
| B | AAEL006783 | histone h3 methyltransferase | 0.989 | 0.595 | Amino Acid Metabolism |
| B | AAEL004401 | peroxinectin | 0.988 | 0.806 | Amino Acid Metabolism |
| B | AAEL002296 | trifunctional enzyme beta subunit (tp-beta) | 0.944 | 0.727 | Amino Acid Metabolism |
| B | AAEL000213 | d-amino acid oxidase | 0.975 | 0.969 | Amino Acid Metabolism |
| B | AAEL005422 | pyrroline-5-carboxylate dehydrogenase | 0.904 | 0.731 | Amino Acid Metabolism |
| B | AAEL008302 | glutamine-dependent nad(+) synthetase | 0.994 | 0.909 | Amino Acid Metabolism |
| B | AAEL005422 | pyrroline-5-carboxylate dehydrogenase | 0.904 | 0.731 | Amino Acid Metabolism |
| B | AAEL015628 | glycine dehydrogenase | 0.661 | 0.944 | Amino Acid Metabolism |
| B | AAEL014426 | glycine dehydrogenase | 0.688 | 0.888 | Amino Acid Metabolism |
| B | AAEL000213 | d-amino acid oxidase | 0.975 | 0.969 | Amino Acid Metabolism |
| B | AAEL000542 | hypothetical protein | 0.819 | 0.861 | Amino Acid Metabolism |
| B | AAEL004025 | glucose dehydrogenase | 0.926 | 0.864 | Amino Acid Metabolism |
| B | AAEL006772 | hypothetical protein | 0.989 | 0.972 | Amino Acid Metabolism |
| B | AAEL000542 | hypothetical protein | 0.819 | 0.861 | Amino Acid Metabolism |
| B | AAEL006772 | hypothetical protein | 0.989 | 0.972 | Amino Acid Metabolism |
| B | AAEL010442 | 4-hydroxyphenylpyruvate dioxygenase | 0.889 | 0.738 | Amino Acid Metabolism |
| B | AAEL006772 | hypothetical protein | 0.989 | 0.972 | Amino Acid Metabolism |
| B | AAEL000542 | hypothetical protein | 0.819 | 0.861 | Amino Acid Metabolism |
| B | AAEL000542 | hypothetical protein | 0.819 | 0.861 | Amino Acid Metabolism |
| B | AAEL010442 | 4-hydroxyphenylpyruvate dioxygenase | 0.889 | 0.738 | Amino Acid Metabolism |
| B | AAEL006772 | hypothetical protein | 0.989 | 0.972 | Amino Acid Metabolism |
| B | AAEL000542 | hypothetical protein | 0.819 | 0.861 | Amino Acid Metabolism |
| B | AAEL000542 | hypothetical protein | 0.819 | 0.861 | Amino Acid Metabolism |
| B | AAEL008930 | protein phosphatase-2b | 0.865 | 0.822 | Cell Growth and Death |
| B | AAEL002277 | camp-dependent protein kinase type i-beta regulatory subunit | 0.989 | 0.623 | Cell Growth and Death |
| B | AAEL000116 | hypothetical protein | 0.979 | 0.770 | Cell Growth and Death |
| B | AAEL002708 | cell division cycle | 0.972 | 0.772 | Cell Growth and Death |
| B | AAEL007241 | hypothetical protein | 0.908 | 0.528 | Cell Growth and Death |
| B | AAEL011376 | mitosis inhibitor protein kinase | 0.973 | 0.731 | Cell Growth and Death |
| B | AAEL010069 | esr1 protein | 0.750 | 0.867 | Cell Growth and Death |
| B | AAEL010967 | tetraspanin, putative | 0.983 | 0.822 | Cell Growth and Death |
| B | AAEL014311 | tetraspanin, putative | 0.986 | 0.921 | Cell Growth and Death |
| B | AAEL006300 | P53 induced protein | 0.991 | 0.620 | Cell Growth and Death |
| B | AAEL007595 | hypothetical protein | 0.498 | 0.898 | Cell Growth and Death |
| B | AAEL010069 | esr1 protein | 0.750 | 0.867 | Cell Growth and Death |
| B | AAEL013596 | phosphatidylinositol 3-kinase regulatory subunit | 0.906 | 0.982 | Cell Growth and Death |
| B | AAEL014900 | ataxia telangiectasia mutated (atm) | 0.732 | 1.000 | Cell Growth and Death |
| B | AAEL009216 | camp-dependent protein kinase type ii regulatory subunit | 0.728 | 0.972 | Cell Growth and Death |
| B | AAEL013310 | hypothetical protein | 0.967 | 0.859 | Cell Growth and Death |
| B | AAEL011596 | mitotic checkpoint serine/threonine-protein kinase bub1 and bubr1 | 0.952 | 0.819 | Cell Growth and Death |
| B | AAEL008123 | hypothetical protein | 0.974 | 0.803 | Cell Growth and Death |
| B | AAEL003744 | hypothetical protein | 0.976 | 0.811 | Cell Growth and Death |
| B | AAEL014900 | ataxia telangiectasia mutated (atm) | 0.732 | 1.000 | Cell Growth and Death |
| B | AAEL004410 | protein phosphatase | 0.996 | 0.910 | Cell Growth and Death |
| B | AAEL011116 | 14-3-3 protein sigma, gamma, zeta, beta/alpha | 0.963 | 0.924 | Cell Growth and Death |
| B | AAEL001407 | cdk4/6 | 0.971 | 0.999 | Cell Growth and Death |
| B | AAEL014900 | ataxia telangiectasia mutated (atm) | 0.732 | 1.000 | Cell Growth and Death |
| B | AAEL001407 | cdk4/6 | 0.971 | 0.999 | Cell Growth and Death |
| B | AAEL010569 | chondroitin synthase | 0.994 | 0.962 | Glycan Biosynthesis and Metabolism |
| B | AAEL007409 | xylosyltransferase | 0.961 | 0.850 | Glycan Biosynthesis and Metabolism |
| B | AAEL007839 | beta-hexosaminidase | 0.997 | 0.950 | Glycan Biosynthesis and Metabolism |
| B | AAEL004402 | alpha-l-iduronidase | 0.694 | 0.815 | Glycan Biosynthesis and Metabolism |
| B | AAEL007839 | beta-hexosaminidase | 0.997 | 0.950 | Glycan Biosynthesis and Metabolism |
| B | AAEL004402 | alpha-l-iduronidase | 0.694 | 0.815 | Glycan Biosynthesis and Metabolism |
| B | AAEL007839 | beta-hexosaminidase | 0.997 | 0.950 | Glycan Biosynthesis and Metabolism |
| B | AAEL007839 | beta-hexosaminidase | 0.997 | 0.950 | Glycan Biosynthesis and Metabolism |
| B | AAEL005460 | alpha-galactosidase/alpha-n-acetylgalactosaminidase | 0.721 | 0.993 | Glycan Biosynthesis and Metabolism |
| B | AAEL011900 | N-acetyllactosaminide beta-1,3-N-acetylglucosaminyltransferase, putative | 0.964 | 0.939 | Glycan Biosynthesis and Metabolism |
| B | AAEL008042 | hypothetical protein | 0.972 | 0.790 | Glycan Biosynthesis and Metabolism |
| B | AAEL002805 | hypothetical protein | 0.960 | 0.748 | Glycan Biosynthesis and Metabolism |
| B | AAEL002483 | hypothetical protein | 0.987 | 0.906 | Glycan Biosynthesis and Metabolism |
| B | AAEL011978 | mannosidase alpha class 2a | 0.935 | 0.995 | Glycan Biosynthesis and Metabolism |
| B | AAEL007839 | beta-hexosaminidase | 0.997 | 0.950 | Glycan Biosynthesis and Metabolism |
| B | AAEL013274 | n-acetylgalactosaminyltransferase | 0.987 | 0.654 | Glycan Biosynthesis and Metabolism |
| B | AAEL012972 | n-acetylgalactosaminyltransferase | 0.938 | 0.780 | Glycan Biosynthesis and Metabolism |
| B | AAEL001121 | n-acetylgalactosaminyltransferase | 0.929 | 0.861 | Glycan Biosynthesis and Metabolism |
| B | AAEL011095 | n-acetylgalactosaminyltransferase | 0.908 | 0.929 | Glycan Biosynthesis and Metabolism |
| B | AAEL009474 | peptidoglycan recognition protein-lc isoform | 0.718 | 0.915 | Glycan Biosynthesis and Metabolism |
| B | AAEL000383 | beta-1,3-galactosyltransferase brn | 0.847 | 0.944 | Glycan Biosynthesis and Metabolism |
| B | AAEL008061 | phosphatidylinositolglycan class N, putative | 0.623 | 0.735 | Glycan Biosynthesis and Metabolism |
| B | AAEL013336 | hypothetical protein | 0.923 | 0.839 | Glycan Biosynthesis and Metabolism |
| B | AAEL003533 | exostosin-1 | 0.878 | 0.945 | Glycan Biosynthesis and Metabolism |
| B | AAEL004916 | hypothetical protein | 0.823 | 0.999 | Glycan Biosynthesis and Metabolism |
| B | AAEL013678 | mannosyl-oligosaccharide alpha-1,2-mannosidase | 0.993 | 0.897 | Glycan Biosynthesis and Metabolism |
| B | AAEL013678 | mannosyl-oligosaccharide alpha-1,2-mannosidase | 0.993 | 0.897 | Glycan Biosynthesis and Metabolism |
| B | AAEL005535 | hypothetical protein | 0.961 | 0.768 | Glycan Biosynthesis and Metabolism |
| B | AAEL012479 | mannosyl-oligosaccharide glucosidase | 0.698 | 0.983 | Glycan Biosynthesis and Metabolism |
| B | AAEL012823 | n-acetylgalactosaminyltransferase | 1.000 | 0.982 | Glycan Biosynthesis and Metabolism |
| B | AAEL008930 | protein phosphatase-2b | 0.865 | 0.822 | Signal Transduction |
| B | AAEL013823 | calcium/calmodulin dependent protein kinase ii | 0.973 | 0.776 | Signal Transduction |
| B | AAEL007931 | P21-activated kinase, pak | 0.902 | 0.894 | Signal Transduction |
| B | AAEL008634 | jnk | 0.994 | 0.581 | Signal Transduction |
| B | AAEL015384 | grb2-associated binder, gab | 0.956 | 0.790 | Signal Transduction |
| B | AAEL013786 | growth factor receptor-bound protein | 0.998 | 0.659 | Signal Transduction |
| B | AAEL013823 | calcium/calmodulin dependent protein kinase ii | 0.973 | 0.776 | Signal Transduction |
| B | AAEL002850 | patched 1, putative | 0.932 | 0.594 | Signal Transduction |
| B | AAEL009869 | low-density lipoprotein receptor (ldl) | 0.901 | 0.616 | Signal Transduction |
| B | AAEL012039 | zinc finger protein | 0.884 | 0.833 | Signal Transduction |
| B | AAEL001876 | decapentaplegic, deca | 0.944 | 0.912 | Signal Transduction |
| B | AAEL013786 | growth factor receptor-bound protein | 0.998 | 0.659 | Signal Transduction |
| B | AAEL002548 | hypothetical protein | 0.820 | 0.712 | Signal Transduction |
| B | AAEL007690 | hypothetical protein | 0.989 | 0.650 | Signal Transduction |
| B | AAEL009775 | rapamycin-insensitive companion of Tor, putative | 0.984 | 0.870 | Signal Transduction |
| B | AAEL006440 | serine/threonine-protein kinase pk61c | 0.939 | 0.715 | Signal Transduction |
| B | AAEL001097 | hypoxia-inducible factor | 0.956 | 0.837 | Signal Transduction |
| B | AAEL005082 | serrate protein | 0.994 | 0.930 | Signal Transduction |
| B | AAEL004336 | hypothetical protein | 0.966 | 0.850 | Signal Transduction |
| B | AAEL010210 | neurogenic locus notch (notch) | 0.935 | 0.649 | Signal Transduction |
| B | AAEL015255 | phosphatidylinositol-4-phosphate 5-kinase type i | 0.969 | 0.748 | Signal Transduction |
| B | AAEL014358 | hypothetical protein | 0.943 | 0.617 | Signal Transduction |
| B | AAEL008510 | sphingosine kinase a, b | 0.999 | 0.628 | Signal Transduction |
| B | AAEL004285 | sphingosine kinase a, b | 0.997 | 0.622 | Signal Transduction |
| B | AAEL014247 | type i inositol-1,4,5-trisphosphate 5-phosphatase | 0.954 | 0.748 | Signal Transduction |
| B | AAEL009294 | phosphatidylinositol 4-kinase | 0.983 | 0.830 | Signal Transduction |
| B | AAEL013891 | type i inositol-1,4,5-trisphosphate 5-phosphatase | 0.844 | 0.966 | Signal Transduction |
| B | AAEL001876 | decapentaplegic, deca | 0.944 | 0.912 | Signal Transduction |
| B | AAEL012835 | 85 kda calcium-independent phospholipase A2 (ipla2) | 0.970 | 0.763 | Signal Transduction |
| B | AAEL008930 | protein phosphatase-2b | 0.865 | 0.822 | Signal Transduction |
| B | AAEL009876 | hypothetical protein | 0.646 | 0.965 | Signal Transduction |
| B | AAEL011359 | NFAT, putative | 0.953 | 0.836 | Signal Transduction |
| B | AAEL008634 | jnk | 0.994 | 0.581 | Signal Transduction |
| B | AAEL003539 | wd-repeat protein | 0.962 | 0.862 | Signal Transduction |
| B | AAEL008930 | protein phosphatase-2b | 0.865 | 0.822 | Signal Transduction |
| B | AAEL013823 | calcium/calmodulin dependent protein kinase ii | 0.973 | 0.776 | Signal Transduction |
| B | AAEL003388 | axis inhibition protein, axin | 0.996 | 0.596 | Signal Transduction |
| B | AAEL009806 | low-density lipoprotein receptor (ldl) | 0.964 | 0.770 | Signal Transduction |
| B | AAEL013920 | disheveled associated activator of morphogenesis | 0.994 | 0.592 | Signal Transduction |
| B | AAEL011359 | NFAT, putative | 0.953 | 0.836 | Signal Transduction |
| B | AAEL013824 | calcium/calmodulin dependent protein kinase ii | 0.981 | 0.844 | Signal Transduction |
| B | AAEL013788 | sodium/calcium exchanger | 0.985 | 0.712 | Signal Transduction |
| C | AAEL012835 | 85 kda calcium-independent phospholipase A2 (ipla2) | 0.970 | 0.763 | Lipid Metabolism |
| C | AAEL009876 | hypothetical protein | 0.646 | 0.965 | Lipid Metabolism |
| C | AAEL000733 | hydroxysteroid dehydrogenase | 0.596 | 0.685 | Lipid Metabolism |
| C | AAEL001586 | glucosyl/glucuronosyl transferases | 0.863 | 0.723 | Lipid Metabolism |
| C | AAEL012835 | 85 kda calcium-independent phospholipase A2 (ipla2) | 0.970 | 0.763 | Lipid Metabolism |
| C | AAEL004870 | cytochrome P450 | 0.963 | 0.572 | Lipid Metabolism |
| C | AAEL009876 | hypothetical protein | 0.646 | 0.965 | Lipid Metabolism |
| C | AAEL002296 | trifunctional enzyme beta subunit (tp-beta) | 0.944 | 0.727 | Lipid Metabolism |
| C | AAEL009311 | 3-hydroxy-3-methylglutaryl-coenzyme A reductase | 0.932 | 0.999 | Lipid Metabolism |
| C | AAEL001740 | candidate tumor suppressor protein | 0.993 | 0.701 | Lipid Metabolism |
| C | AAEL008740 | synaptic glycoprotein sc2 | 0.828 | 0.952 | Lipid Metabolism |
| C | AAEL000733 | hydroxysteroid dehydrogenase | 0.596 | 0.685 | Lipid Metabolism |
| C | AAEL012835 | 85 kda calcium-independent phospholipase A2 (ipla2) | 0.970 | 0.763 | Lipid Metabolism |
| C | AAEL009876 | hypothetical protein | 0.646 | 0.965 | Lipid Metabolism |
| C | AAEL007793 | alkyldihydroxyacetonephosphate synthase | 0.997 | 0.918 | Lipid Metabolism |
| C | AAEL002296 | trifunctional enzyme beta subunit (tp-beta) | 0.944 | 0.727 | Lipid Metabolism |
| C | AAEL002296 | trifunctional enzyme beta subunit (tp-beta) | 0.944 | 0.727 | Lipid Metabolism |
| C | AAEL005458 | carnitine o-acyltransferase | 0.992 | 0.711 | Lipid Metabolism |
| C | AAEL006966 | vitellogenin, putative | 0.815 | 0.778 | Lipid Metabolism |
| C | AAEL008510 | sphingosine kinase a, b | 0.999 | 0.628 | Lipid Metabolism |
| C | AAEL011697 | glycerol kinase | 0.989 | 0.606 | Lipid Metabolism |
| C | AAEL004285 | sphingosine kinase a, b | 0.997 | 0.622 | Lipid Metabolism |
| C | AAEL004970 | hypothetical protein | 0.889 | 0.963 | Lipid Metabolism |
| C | AAEL012340 | lipase 1 precursor | 0.857 | 0.944 | Lipid Metabolism |
| C | AAEL012835 | 85 kda calcium-independent phospholipase A2 (ipla2) | 0.970 | 0.763 | Lipid Metabolism |
| C | AAEL014198 | cdp-diacylglycerol--glycerol-3-phosphate 3-phosphatidyltransferase | 0.963 | 0.606 | Lipid Metabolism |
| C | AAEL008510 | sphingosine kinase a, b | 0.999 | 0.628 | Lipid Metabolism |
| C | AAEL004285 | sphingosine kinase a, b | 0.997 | 0.622 | Lipid Metabolism |
| C | AAEL009876 | hypothetical protein | 0.646 | 0.965 | Lipid Metabolism |
| C | AAEL012835 | 85 kda calcium-independent phospholipase A2 (ipla2) | 0.970 | 0.763 | Lipid Metabolism |
| C | AAEL009876 | hypothetical protein | 0.646 | 0.965 | Lipid Metabolism |
| C | AAEL000634 | ceramide glucosyltransferase | 0.995 | 0.618 | Lipid Metabolism |
| C | AAEL003402 | sphingomyelin phosphodiesterase | 0.949 | 0.589 | Lipid Metabolism |
| C | AAEL000735 | acyl-CoA oxidase | 0.959 | 0.850 | Lipid Metabolism |
| C | AAEL008841 | acyl-CoA oxidase | 0.770 | 0.975 | Lipid Metabolism |
| C | AAEL002287 | trans-prenyltransferase | 0.983 | 0.900 | Lipid Metabolism |
| C | AAEL000735 | acyl-CoA oxidase | 0.959 | 0.850 | Lipid Metabolism |
| C | AAEL008841 | acyl-CoA oxidase | 0.770 | 0.975 | Lipid Metabolism |
| C | AAEL014026 | 1-acylglycerol-3-phosphate acyltransferase | 0.941 | 0.716 | Lipid Metabolism |
| C | AAEL000735 | acyl-CoA oxidase | 0.959 | 0.850 | Lipid Metabolism |
| C | AAEL008841 | acyl-CoA oxidase | 0.770 | 0.975 | Lipid Metabolism |
| C | AAEL014026 | 1-acylglycerol-3-phosphate acyltransferase | 0.941 | 0.716 | Lipid Metabolism |
| C | AAEL006772 | hypothetical protein | 0.989 | 0.972 | Lipid Metabolism |
| C | AAEL014026 | 1-acylglycerol-3-phosphate acyltransferase | 0.941 | 0.716 | Lipid Metabolism |
| C | AAEL012494 | cytochrome P450 | 0.531 | 0.869 | Lipid Metabolism |
| C | AAEL006024 | Vanin-like protein 2 precursor, putative | 0.969 | 0.782 | Metabolism of Cofactors and Vitamins |
| C | AAEL011744 | DEAD box ATP-dependent RNA helicase | 0.960 | 0.953 | Metabolism of Cofactors and Vitamins |
| C | AAEL011187 | U520 | 0.969 | 0.637 | Metabolism of Cofactors and Vitamins |
| C | AAEL008788 | es2 protein | 0.955 | 0.843 | Metabolism of Cofactors and Vitamins |
| C | AAEL002528 | histone deacetylase | 0.989 | 0.642 | Metabolism of Cofactors and Vitamins |
| C | AAEL011597 | hypothetical protein | 0.967 | 0.931 | Metabolism of Cofactors and Vitamins |
| C | AAEL000278 | poly(p)/atp nad kinase | 0.972 | 0.753 | Metabolism of Cofactors and Vitamins |
| C | AAEL003083 | myotubularin | 0.816 | 0.914 | Metabolism of Cofactors and Vitamins |
| C | AAEL010276 | aminomethyltransferase | 0.694 | 0.645 | Metabolism of Cofactors and Vitamins |
| C | AAEL008925 | 5-formyltetrahydrofolate cyclo-ligase | 0.894 | 0.817 | Metabolism of Cofactors and Vitamins |
| C | AAEL010694 | 5-formyltetrahydrofolate cyclo-ligase | 0.905 | 0.798 | Metabolism of Cofactors and Vitamins |
| C | AAEL002528 | histone deacetylase | 0.989 | 0.642 | Metabolism of Cofactors and Vitamins |
| C | AAEL001586 | glucosyl/glucuronosyl transferases | 0.863 | 0.723 | Metabolism of Cofactors and Vitamins |
| C | AAEL001978 | uroporphyrinogen iii synthase | 0.970 | 0.817 | Metabolism of Cofactors and Vitamins |
| C | AAEL001586 | glucosyl/glucuronosyl transferases | 0.863 | 0.723 | Metabolism of Cofactors and Vitamins |
| C | AAEL011597 | hypothetical protein | 0.967 | 0.931 | Metabolism of Cofactors and Vitamins |
| C | AAEL014842 | multiple inositol polyphosphate phosphatase | 0.879 | 0.699 | Metabolism of Cofactors and Vitamins |
| C | AAEL015040 | multiple inositol polyphosphate phosphatase | 0.824 | 0.786 | Metabolism of Cofactors and Vitamins |
| C | AAEL003083 | myotubularin | 0.816 | 0.914 | Metabolism of Cofactors and Vitamins |
| C | AAEL011597 | hypothetical protein | 0.967 | 0.931 | Metabolism of Cofactors and Vitamins |
| C | AAEL014506 | hypothetical protein | 0.744 | 0.984 | Metabolism of Cofactors and Vitamins |
| C | AAEL003083 | myotubularin | 0.816 | 0.914 | Metabolism of Cofactors and Vitamins |
| C | AAEL015392 | hypothetical protein | 0.807 | 0.820 | Metabolism of Cofactors and Vitamins |
| C | AAEL005744 | DEAD box ATP-dependent RNA helicase | 0.989 | 0.902 | Metabolism of Cofactors and Vitamins |
| C | AAEL009970 | salivary apyrase, putative | 0.999 | 0.964 | Metabolism of Cofactors and Vitamins |
| C | AAEL002269 | purine nucleoside phosphorylase | 0.664 | 0.863 | Metabolism of Cofactors and Vitamins |
| C | AAEL001069 | histone deacetylase | 0.978 | 0.862 | Metabolism of Cofactors and Vitamins |
| C | AAEL008302 | glutamine-dependent nad(+) synthetase | 0.994 | 0.909 | Metabolism of Cofactors and Vitamins |
| C | AAEL001069 | histone deacetylase | 0.978 | 0.862 | Metabolism of Cofactors and Vitamins |
| C | AAEL007633 | dihydropyrimidinase | 0.576 | 0.849 | Metabolism of Cofactors and Vitamins |
| C | AAEL015171 | cornichon | 0.786 | 0.901 | Metabolism of Cofactors and Vitamins |
| C | AAEL012494 | cytochrome P450 | 0.531 | 0.869 | Metabolism of Cofactors and Vitamins |
| C | AAEL004494 | hypothetical protein | 0.545 | 0.935 | Metabolism of Cofactors and Vitamins |
| C | AAEL008320 | hypothetical protein | 0.983 | 0.985 | Metabolism of Cofactors and Vitamins |
| C | AAEL002194 | uricase | 0.651 | 0.849 | Nucleotide Metabolism |
| C | AAEL012700 | ATP-binding cassette sub-family A member 3, putative | 0.782 | 0.816 | Nucleotide Metabolism |
| C | AAEL009022 | adenylate cyclase type | 0.991 | 0.633 | Nucleotide Metabolism |
| C | AAEL014178 | DNA polymerase delta catalytic subunit | 0.990 | 0.781 | Nucleotide Metabolism |
| C | AAEL002230 | chromodomain helicase DNA binding protein | 0.968 | 0.750 | Nucleotide Metabolism |
| C | AAEL008463 | DEAD box ATP-dependent RNA helicase | 0.901 | 0.810 | Nucleotide Metabolism |
| C | AAEL009937 | calcium/calmodulin-dependent serine protein kinase membrane-associated guanylate kinase (cask) | 0.953 | 0.767 | Nucleotide Metabolism |
| C | AAEL015410 | AMP deaminase | 0.729 | 0.859 | Nucleotide Metabolism |
| C | AAEL011649 | DNA polymerase theta | 0.961 | 0.756 | Nucleotide Metabolism |
| C | AAEL006184 | hypothetical protein | 0.960 | 0.784 | Nucleotide Metabolism |
| C | AAEL001629 | camp-specific 3,5-cyclic phosphodiesterase | 0.965 | 0.922 | Nucleotide Metabolism |
| C | AAEL013473 | camp-specific 3,5-cyclic phosphodiesterase | 0.982 | 0.563 | Nucleotide Metabolism |
| C | AAEL013372 | abc transporter | 0.801 | 0.983 | Nucleotide Metabolism |
| C | AAEL004954 | hypothetical protein | 0.913 | 0.877 | Nucleotide Metabolism |
| C | AAEL009618 | splicing endonuclease positive effector sen1 | 0.878 | 0.911 | Nucleotide Metabolism |
| C | AAEL000345 | membrane associated guanylate kinase inverted 1, magi1 | 0.907 | 0.967 | Nucleotide Metabolism |
| C | AAEL014831 | RNA polymerase II subunit Rpb10, putative | 0.887 | 0.884 | Nucleotide Metabolism |
| C | AAEL011265 | abc transporter | 0.946 | 0.947 | Nucleotide Metabolism |
| C | AAEL005330 | atrial natriuretic peptide receptor | 0.954 | 0.932 | Nucleotide Metabolism |
| C | AAEL014506 | hypothetical protein | 0.744 | 0.984 | Nucleotide Metabolism |
| C | AAEL012922 | unconventional myosin 95e isoform | 0.743 | 0.980 | Nucleotide Metabolism |
| C | AAEL014178 | DNA polymerase delta catalytic subunit | 0.990 | 0.781 | Nucleotide Metabolism |
| C | AAEL012973 | uracil phosphoribosyltransferase | 0.967 | 0.841 | Nucleotide Metabolism |
| C | AAEL005839 | uridine phosphorylase | 0.618 | 0.848 | Nucleotide Metabolism |
| C | AAEL000629 | adenylate kinase 3, putative | 0.996 | 0.911 | Nucleotide Metabolism |
| C | AAEL014831 | RNA polymerase II subunit Rpb10, putative | 0.887 | 0.884 | Nucleotide Metabolism |
| C | AAEL009490 | carbamoyl-phosphate synthase large chain | 0.916 | 0.899 | Nucleotide Metabolism |
| C | AAEL009970 | salivary apyrase, putative | 0.999 | 0.964 | Nucleotide Metabolism |
| C | AAEL002269 | purine nucleoside phosphorylase | 0.664 | 0.863 | Nucleotide Metabolism |
| C | AAEL004942 | helicase | 0.961 | 0.788 | Nucleotide Metabolism |
| C | AAEL013235 | ATP-dependent RNA helicase | 0.939 | 0.906 | Nucleotide Metabolism |
| C | AAEL001719 | ATP-dependent RNA helicase | 0.838 | 0.943 | Nucleotide Metabolism |
| C | AAEL010443 | membrane associated guanylate kinase inverted 1, magi1 | 0.989 | 0.865 | Nucleotide Metabolism |
| C | AAEL004494 | hypothetical protein | 0.545 | 0.935 | Nucleotide Metabolism |
| C | AAEL008320 | hypothetical protein | 0.983 | 0.985 | Nucleotide Metabolism |
| C | AAEL012294 | adenylate cyclase type ix | 0.991 | 0.952 | Nucleotide Metabolism |
| C | AAEL004698 | DNA primase large subunit | 0.969 | 0.811 | Nucleotide Metabolism |
| C | AAEL011098 | hypothetical protein | 0.635 | 0.957 | Nucleotide Metabolism |
| C | AAEL013900 | camp and camp-inhibited cgmp 3,5-cyclic phosphodiesterase | 0.640 | 0.820 | Nucleotide Metabolism |
| C | AAEL005177 | adenylate cyclase | 0.999 | 0.638 | Nucleotide Metabolism |
| C | AAEL009970 | salivary apyrase, putative | 0.999 | 0.964 | Nucleotide Metabolism |
| C | AAEL002269 | purine nucleoside phosphorylase | 0.664 | 0.863 | Nucleotide Metabolism |
| C | AAEL007633 | dihydropyrimidinase | 0.576 | 0.849 | Nucleotide Metabolism |
| C | AAEL001242 | ribosomal pseudouridine synthase | 0.953 | 0.825 | Nucleotide Metabolism |
| C | AAEL006033 | dihydroorotate dehydrogenase | 0.809 | 0.940 | Nucleotide Metabolism |
| C | AAEL004698 | DNA primase large subunit | 0.969 | 0.811 | Nucleotide Metabolism |
| C | AAEL011098 | hypothetical protein | 0.635 | 0.957 | Nucleotide Metabolism |
| D | AAEL003961 | short-chain dehydrogenase | 0.968 | 0.748 | Xenobiotics Biodegradation and Metabolism |
| D | AAEL003961 | short-chain dehydrogenase | 0.968 | 0.748 | Xenobiotics Biodegradation and Metabolism |
| D | AAEL003961 | short-chain dehydrogenase | 0.968 | 0.748 | Xenobiotics Biodegradation and Metabolism |
| D | AAEL002296 | trifunctional enzyme beta subunit (tp-beta) | 0.944 | 0.727 | Xenobiotics Biodegradation and Metabolism |
| D | AAEL003961 | short-chain dehydrogenase | 0.968 | 0.748 | Xenobiotics Biodegradation and Metabolism |
| D | AAEL001586 | glucosyl/glucuronosyl transferases | 0.863 | 0.723 | Xenobiotics Biodegradation and Metabolism |
| D | AAEL005839 | uridine phosphorylase | 0.618 | 0.848 | Xenobiotics Biodegradation and Metabolism |
| D | AAEL000918 | carboxylesterase | 0.932 | 0.968 | Xenobiotics Biodegradation and Metabolism |
| D | AAEL014303 | neuroligin, putative | 0.846 | 0.955 | Xenobiotics Biodegradation and Metabolism |
| D | AAEL007808 | cytochrome P450 | 0.678 | 0.704 | Xenobiotics Biodegradation and Metabolism |
| D | AAEL014412 | cytochrome P450 | 0.535 | 0.582 | Xenobiotics Biodegradation and Metabolism |
| D | AAEL007677 | phospholysine phosphohistidine inorganic pyrophosphate phosphatase | 0.945 | 0.932 | Xenobiotics Biodegradation and Metabolism |
| D | AAEL014842 | multiple inositol polyphosphate phosphatase | 0.879 | 0.699 | Xenobiotics Biodegradation and Metabolism |
| D | AAEL015040 | multiple inositol polyphosphate phosphatase | 0.824 | 0.786 | Xenobiotics Biodegradation and Metabolism |
| D | AAEL003961 | short-chain dehydrogenase | 0.968 | 0.748 | Xenobiotics Biodegradation and Metabolism |
| D | AAEL002296 | trifunctional enzyme beta subunit (tp-beta) | 0.944 | 0.727 | Xenobiotics Biodegradation and Metabolism |
| D | AAEL007808 | cytochrome P450 | 0.678 | 0.704 | Xenobiotics Biodegradation and Metabolism |
| D | AAEL014412 | cytochrome P450 | 0.535 | 0.582 | Xenobiotics Biodegradation and Metabolism |
| D | AAEL003961 | short-chain dehydrogenase | 0.968 | 0.748 | Xenobiotics Biodegradation and Metabolism |
| D | AAEL006772 | hypothetical protein | 0.989 | 0.972 | Xenobiotics Biodegradation and Metabolism |
| D | AAEL006772 | hypothetical protein | 0.989 | 0.972 | Xenobiotics Biodegradation and Metabolism |
| D | AAEL007633 | dihydropyrimidinase | 0.576 | 0.849 | Xenobiotics Biodegradation and Metabolism |
| D | AAEL012494 | cytochrome P450 | 0.531 | 0.869 | Xenobiotics Biodegradation and Metabolism |
| D | AAEL006772 | hypothetical protein | 0.989 | 0.972 | Xenobiotics Biodegradation and Metabolism |
| D | AAEL012494 | cytochrome P450 | 0.531 | 0.869 | Xenobiotics Biodegradation and Metabolism |
| D | AAEL009131 | cytochrome P450 | 0.959 | 0.732 | Xenobiotics Biodegradation and Metabolism |
| D | AAEL014891 | cytochrome P450 | 0.829 | 0.901 | Xenobiotics Biodegradation and Metabolism |
| D | AAEL009762 | cytochrome P450 | 0.649 | 0.884 | Xenobiotics Biodegradation and Metabolism |
| D | AAEL009131 | cytochrome P450 | 0.959 | 0.732 | Xenobiotics Biodegradation and Metabolism |
| D | AAEL014891 | cytochrome P450 | 0.829 | 0.901 | Xenobiotics Biodegradation and Metabolism |
| D | AAEL009762 | cytochrome P450 | 0.649 | 0.884 | Xenobiotics Biodegradation and Metabolism |
| E | AAEL000918 | carboxylesterase | 0.932 | 0.968 | Biosynthesis of Secondary Metabolites |
| E | AAEL014303 | neuroligin, putative | 0.846 | 0.955 | Biosynthesis of Secondary Metabolites |
| E | AAEL002194 | uricase | 0.651 | 0.849 | Biosynthesis of Secondary Metabolites |
| E | AAEL007808 | cytochrome P450 | 0.678 | 0.704 | Biosynthesis of Secondary Metabolites |
| E | AAEL014412 | cytochrome P450 | 0.535 | 0.582 | Biosynthesis of Secondary Metabolites |
| E | AAEL003961 | short-chain dehydrogenase | 0.968 | 0.748 | Biosynthesis of Secondary Metabolites |
| E | AAEL007808 | cytochrome P450 | 0.678 | 0.704 | Biosynthesis of Secondary Metabolites |
| E | AAEL014412 | cytochrome P450 | 0.535 | 0.582 | Biosynthesis of Secondary Metabolites |
| E | AAEL004401 | peroxinectin | 0.988 | 0.806 | Biosynthesis of Secondary Metabolites |
| E | AAEL006772 | hypothetical protein | 0.989 | 0.972 | Biosynthesis of Secondary Metabolites |
| E | AAEL004888 | cytochrome P450 | 0.704 | 0.769 | Biosynthesis of Secondary Metabolites |
| E | AAEL015655 | cytochrome P450 | 0.898 | 0.769 | Biosynthesis of Secondary Metabolites |
| E | AAEL006772 | hypothetical protein | 0.989 | 0.972 | Biosynthesis of Secondary Metabolites |
| E | AAEL009131 | cytochrome P450 | 0.959 | 0.732 | Biosynthesis of Secondary Metabolites |
| E | AAEL014891 | cytochrome P450 | 0.829 | 0.901 | Biosynthesis of Secondary Metabolites |
| E | AAEL009762 | cytochrome P450 | 0.649 | 0.884 | Biosynthesis of Secondary Metabolites |
| E | AAEL000213 | d-amino acid oxidase | 0.975 | 0.969 | Biosynthesis of Secondary Metabolites |
| E | AAEL009131 | cytochrome P450 | 0.959 | 0.732 | Biosynthesis of Secondary Metabolites |
| E | AAEL001478 | bile acid beta-glucosidase, putative | 0.917 | 0.918 | Biosynthesis of Secondary Metabolites |
| E | AAEL014891 | cytochrome P450 | 0.829 | 0.901 | Biosynthesis of Secondary Metabolites |
| E | AAEL009762 | cytochrome P450 | 0.649 | 0.884 | Biosynthesis of Secondary Metabolites |
| E | AAEL004401 | peroxinectin | 0.988 | 0.806 | Energy Metabolism |
| E | AAEL010276 | aminomethyltransferase | 0.694 | 0.645 | Energy Metabolism |
| E | AAEL005520 | carbonic anhydrase | 0.974 | 0.768 | Energy Metabolism |
| E | AAEL014491 | sin3b | 0.993 | 0.613 | Energy Metabolism |
| E | AAEL013334 | hypothetical protein | 0.998 | 0.710 | Energy Metabolism |
| E | AAEL009903 | hypothetical protein | 0.903 | 0.849 | Energy Metabolism |
| E | AAEL000321 | acetyl-coa synthetase | 0.940 | 0.596 | Energy Metabolism |
| E | AAEL010505 | ribulose-5-phosphate-3-epimerase | 0.983 | 0.573 | Energy Metabolism |
| E | AAEL008031 | carbonic anhydrase | 0.894 | 0.984 | Energy Metabolism |
| E | AAEL004930 | carbonic anhydrase | 0.840 | 0.906 | Energy Metabolism |
| E | AAEL005508 | NADH-ubiquinone oxidoreductase 24 kda subunit | 0.915 | 0.950 | Energy Metabolism |
| E | AAEL009257 | hypothetical protein | 0.986 | 0.976 | Energy Metabolism |
| E | AAEL005143 | ubiquinone binding protein, putative | 0.920 | 0.799 | Energy Metabolism |
| E | AAEL009964 | hypothetical protein | 0.983 | 0.964 | Energy Metabolism |
| E | AAEL000986 | NADH-ubiquinone oxidoreductase ashi subunit | 0.782 | 0.947 | Energy Metabolism |
| E | AAEL008490 | NADH dehydrogenase, putative | 0.985 | 0.982 | Energy Metabolism |
| E | AAEL002037 | hypothetical protein | 0.969 | 0.708 | Energy Metabolism |
| E | AAEL001230 | myelin transcription factor 1, myt1 | 0.963 | 0.827 | Energy Metabolism |
| E | AAEL012747 | hypothetical protein | 0.951 | 0.841 | Energy Metabolism |
| E | AAEL008787 | ATP synthase alpha subunit vacuolar | 0.935 | 0.703 | Energy Metabolism |
| E | AAEL000291 | Vacuolar ATP synthase 16 kDa proteolipid subunit | 0.931 | 0.914 | Energy Metabolism |
| E | AAEL012035 | vacuolar ATP synthase subunit e | 0.998 | 0.703 | Energy Metabolism |
| E | AAEL005798 | ATP synthase subunit beta vacuolar | 0.983 | 0.731 | Energy Metabolism |
| E | AAEL014178 | DNA polymerase delta catalytic subunit | 0.990 | 0.781 | Replication and Repair |
| E | AAEL003906 | endonuclease iii | 0.976 | 0.857 | Replication and Repair |
| E | AAEL014178 | DNA polymerase delta catalytic subunit | 0.990 | 0.781 | Replication and Repair |
| E | AAEL014178 | DNA polymerase delta catalytic subunit | 0.990 | 0.781 | Replication and Repair |
| E | AAEL014856 | DNA mismatch repair protein spellchecker 1 | 0.907 | 0.908 | Replication and Repair |
| E | AAEL014178 | DNA polymerase delta catalytic subunit | 0.990 | 0.781 | Replication and Repair |
| E | AAEL013205 | rad25/xp-b DNA repair helicase | 0.893 | 0.564 | Replication and Repair |
| E | AAEL012750 | transcription factor TFIIH-subunit, putative | 0.809 | 0.724 | Replication and Repair |
| E | AAEL008148 | 8-oxoguanine DNA glycosylase | 0.902 | 0.870 | Replication and Repair |
| E | AAEL013179 | 8-oxoguanine DNA glycosylase | 0.892 | 0.979 | Replication and Repair |
| E | AAEL001324 | replication factor C large subunit, putative | 0.977 | 0.672 | Replication and Repair |
| E | AAEL009465 | replication factor c / DNA polymerase iii gamma-tau subunit | 0.999 | 0.944 | Replication and Repair |
| E | AAEL004698 | DNA primase large subunit | 0.969 | 0.811 | Replication and Repair |
| E | AAEL013690 | DNA mismatch repair protein pms2 | 0.958 | 0.699 | Replication and Repair |
| E | AAEL010033 | DNA mismatch repair protein pms2 | 0.954 | 0.738 | Replication and Repair |
| E | AAEL001324 | replication factor C large subunit, putative | 0.977 | 0.672 | Replication and Repair |
| E | AAEL009465 | replication factor c / DNA polymerase iii gamma-tau subunit | 0.999 | 0.944 | Replication and Repair |
| E | AAEL001324 | replication factor C large subunit, putative | 0.977 | 0.672 | Replication and Repair |
| E | AAEL003897 | DNA repair protein xp-c / rad4 | 0.998 | 0.947 | Replication and Repair |
| E | AAEL009465 | replication factor c / DNA polymerase iii gamma-tau subunit | 0.999 | 0.944 | Replication and Repair |
| E | AAEL002098 | DNA repair endonuclease xp-f / mei-9 / rad1 | 0.432 | 0.940 | Replication and Repair |
| G | AAEL001165 | ras GTP exchange factor, son of sevenless | 0.952 | 0.982 | Signal Transduction |
| G | AAEL013596 | phosphatidylinositol 3-kinase regulatory subunit | 0.906 | 0.982 | Signal Transduction |
| G | AAEL013824 | calcium/calmodulin dependent protein kinase ii | 0.981 | 0.844 | Signal Transduction |
| G | AAEL009739 | cbl-d | 0.980 | 0.825 | Signal Transduction |
| G | AAEL000693 | fkbp-rapamycin associated protein | 0.991 | 0.960 | Signal Transduction |
| G | AAEL001864 | Eukaryotic translation initiation factor 4E binding protein (4EBP) | 0.981 | 0.718 | Signal Transduction |
| G | AAEL001165 | ras GTP exchange factor, son of sevenless | 0.952 | 0.982 | Signal Transduction |
| G | AAEL013596 | phosphatidylinositol 3-kinase regulatory subunit | 0.906 | 0.982 | Signal Transduction |
| G | AAEL009739 | cbl-d | 0.980 | 0.825 | Signal Transduction |
| G | AAEL000393 | suppressors of cytokine signalling | 0.995 | 0.894 | Signal Transduction |
| G | AAEL012553 | tyrosine protein kinase | 0.757 | 0.951 | Signal Transduction |
| G | AAEL003532 | hypothetical protein | 0.801 | 0.805 | Signal Transduction |
| G | AAEL014510 | sprouty | 0.999 | 0.824 | Signal Transduction |
| G | AAEL013596 | phosphatidylinositol 3-kinase regulatory subunit | 0.906 | 0.982 | Signal Transduction |
| G | AAEL005943 | ser/thr kinase stk11 (lkb1) | 0.998 | 0.961 | Signal Transduction |
| G | AAEL000693 | fkbp-rapamycin associated protein | 0.991 | 0.960 | Signal Transduction |
| G | AAEL001864 | Eukaryotic translation initiation factor 4E binding protein (4EBP) | 0.981 | 0.718 | Signal Transduction |
| G | AAEL008234 | dishevelled | 0.973 | 0.807 | Signal Transduction |
| G | AAEL010433 | groucho protein (enhancer of split) | 0.998 | 0.983 | Signal Transduction |
| G | AAEL002253 | beta-1,3-n-acetylglucosaminyltransferase radical fringe (o-fucosylpeptide 3-beta-n-acetylglucosaminyltransferase) | 0.991 | 0.873 | Signal Transduction |
| G | AAEL008617 | hypothetical protein | 0.961 | 0.752 | Signal Transduction |
| G | AAEL001476 | hypothetical protein | 0.897 | 0.819 | Signal Transduction |
| G | AAEL013596 | phosphatidylinositol 3-kinase regulatory subunit | 0.906 | 0.982 | Signal Transduction |
| G | AAEL001711 | activin receptor type I, putative | 0.975 | 0.868 | Signal Transduction |
| G | AAEL009652 | activin receptor type ii | 0.988 | 0.876 | Signal Transduction |
| G | AAEL013596 | phosphatidylinositol 3-kinase regulatory subunit | 0.906 | 0.982 | Signal Transduction |
| G | AAEL009732 | rac gtpase | 0.953 | 0.893 | Signal Transduction |
| G | AAEL004797 | serine/threonine kinase NLK | 0.969 | 0.716 | Signal Transduction |
| G | AAEL006778 | frizzled | 0.990 | 0.875 | Signal Transduction |
| G | AAEL013824 | calcium/calmodulin dependent protein kinase ii | 0.981 | 0.844 | Signal Transduction |
| G | AAEL008234 | dishevelled | 0.973 | 0.807 | Signal Transduction |
| G | AAEL010433 | groucho protein (enhancer of split) | 0.998 | 0.983 | Signal Transduction |
| G | AAEL009732 | rac gtpase | 0.953 | 0.893 | Signal Transduction |
| G | AAEL000993 | dally | 0.922 | 0.817 | Signal Transduction |
| G | AAEL000815 | hypothetical protein | 0.983 | 0.801 | Sorting and Degradation |
| G | AAEL010516 | hypothetical protein | 0.988 | 0.868 | Sorting and Degradation |
| G | AAEL006910 | ubiquitination factor E4 | 0.992 | 0.627 | Sorting and Degradation |
| G | AAEL007353 | cullin | 0.976 | 0.839 | Sorting and Degradation |
| G | AAEL015199 | wd-repeat protein | 0.980 | 0.802 | Sorting and Degradation |
| G | AAEL012435 | tripartite motif protein | 0.969 | 0.762 | Sorting and Degradation |
| G | AAEL000116 | hypothetical protein | 0.979 | 0.770 | Sorting and Degradation |
| G | AAEL002708 | cell division cycle | 0.972 | 0.772 | Sorting and Degradation |
| G | AAEL003907 | ubiquitination factor E4a | 0.939 | 0.743 | Sorting and Degradation |
| G | AAEL007241 | hypothetical protein | 0.908 | 0.528 | Sorting and Degradation |
| G | AAEL000758 | ubiquitin-activating enzyme E1 | 0.927 | 0.948 | Sorting and Degradation |
| G | AAEL002306 | hect E3 ubiquitin ligase | 0.855 | 0.946 | Sorting and Degradation |
| G | AAEL002906 | 26S proteasome regulatory subunit rpn2 | 0.995 | 0.983 | Sorting and Degradation |
| G | AAEL011834 | hypothetical protein | 0.926 | 0.992 | Sorting and Degradation |
| G | AAEL008374 | E3 ubiquitin-protein ligase nedd-4 | 0.965 | 0.776 | Sorting and Degradation |
| G | AAEL006291 | cullin | 0.998 | 0.923 | Sorting and Degradation |
| G | AAEL006413 | ubiquitin-conjugating enzyme morgue | 0.974 | 0.867 | Sorting and Degradation |
| G | AAEL009739 | cbl-d | 0.980 | 0.825 | Sorting and Degradation |
| G | AAEL012500 | ubiquitin-protein ligase | 0.986 | 0.951 | Sorting and Degradation |
| G | AAEL013454 | ubiquitin protein ligase | 0.867 | 0.920 | Sorting and Degradation |
| G | AAEL003271 | hypothetical protein | 0.892 | 0.956 | Sorting and Degradation |
| G | AAEL003744 | hypothetical protein | 0.976 | 0.811 | Sorting and Degradation |
