# Supplementary material for: Global Cross-Talk of Genes of the Mosquito Aedes aegypti in Response to Dengue Virus Infection
Source: PLoS Negl Trop Dis. 2011 Nov 15;5(11):e1385. doi: 10.1371/journal.pntd.0001385 (PMC3216916; doi:10.1371/journal.pntd.0001385)
Supplement: Table S3 — List of genes and primer sequences (5′-3′) used for qRT-PCR validations. (DOCX) [file pntd.0001385.s004.docx]

Table S3. List of genes and primer sequences (5’-3’) used for qRT-PCR validations.

__________________________________________________________________

Gene ID Forward Primer Reverse Primer

__________________________________________________________________

AAEL004175* AGAAGTGGCCATCATTCCAA GATACCACGGACCTGGGAGT

AAEL003034^a^ CAGTGCCAAAAGTTGAAGGA CATCCGGGATACAAGAATGG

AAEL013284^b^ CCACTGTTCGCTACGAATGA GCGGTCGGTAAAGGTATCAC

AAEL006956^c^ CGGAATCAACACTTGGAGGT TGACACCATTTGGCATCGTA

AAEL007498^d^ CAATACCATTTACCGAATCCA CGCTGGTAGGCATCTTTGAT

AAEL001182^e^ CTGCAGGGAACGAAACAGAG TGATCAACGGGAGGAGTTTC

____________________________________________________________

* Ribosomal protein gene S17 used as control, ^a^ HSP20-like chaperone, ^b^ serine-type enodpeptidase, ^c^ suppressor of ty, ^d^ conserved hypothetical protein and ^e^ phosphotyrosyl phosphatase activator.
