# Supplementary material for: Global Cross-Talk of Genes of the Mosquito Aedes aegypti in Response to Dengue Virus Infection
Source: PLoS Negl Trop Dis. 2011 Nov 15;5(11):e1385. doi: 10.1371/journal.pntd.0001385 (PMC3216916; doi:10.1371/journal.pntd.0001385)
Supplement: Table S4 — List of significant DENV responsive genes that showed higher fold-change than the majority of genes belonging to the predicted modules wherein correlated expression patterns were observed. (DOCX) [file pntd.0001385.s005.docx]

Table S4. List of significant DENV responsive genes that showed higher fold-change than the majority of genes belonging to the predicted modules wherein correlated expression patterns were observed. N/A = not associated with a particular module.

| Responsive Gene | Fold  change | Module |
| --- | --- | --- |
| AAEL014403 | 6.2 | N/A |
| AAEL004197 | 4.3 | N/A |
| AAEL007617 | 4.2 | N/A |
| AAEL001436 | 3.8 | N/A |
| AAEL004651 | 3.8 | N/A |
| AAEL001097 | 3.6 | N/A |
| AAEL001097 | 3.6 | B |
| AAEL006492 | 3.5 | N/A |
| AAEL007183 | 3.5 | N/A |
| AAEL015231 | 3.5 | N/A |
| AAEL001467 | 3.4 | N/A |
| AAEL007234 | 3.2 | N/A |
| AAEL013063 | 3.1 | N/A |
| AAEL015125 | 3.1 | N/A |
| AAEL001867 | 3.0 | N/A |
| AAEL005791 | 3.0 | N/A |
| AAEL007953 | 3.0 | N/A |
| AAEL007985 | 3.0 | N/A |
| AAEL012756 | 3.0 | N/A |
| AAEL014021 | 3.0 | N/A |
| AAEL014164 | 3.0 | N/A |
| AAEL014223 | 3.0 | N/A |
| AAEL001460 | 2.9 | N/A |
| AAEL005447 | 2.9 | N/A |
| AAEL010720 | 2.9 | N/A |
| AAEL011065 | 2.9 | N/A |
| AAEL013104 | 2.9 | N/A |
| AAEL004704 | 2.8 | N/A |
| AAEL004954 | 2.8 | N/A |
| AAEL008555 | 2.8 | N/A |
| AAEL008845 | 2.8 | N/A |
| AAEL009762 | 2.8 | N/A |
| AAEL012603 | 2.8 | N/A |
| AAEL012604 | 2.8 | N/A |
| AAEL013178 | 2.8 | N/A |
| AAEL014362 | 2.8 | N/A |
| AAEL004954 | 2.8 | C |
| AAEL009762 | 2.8 | D |
| AAEL009762 | 2.8 | D |
| AAEL009762 | 2.8 | E |
| AAEL009762 | 2.8 | E |
| AAEL002008 | 2.7 | N/A |
| AAEL003191 | 2.7 | N/A |
| AAEL006127 | 2.7 | N/A |
| AAEL008054 | 2.7 | N/A |
| AAEL010701 | 2.7 | N/A |
| AAEL011430 | 2.7 | N/A |
| AAEL014026 | 2.7 | N/A |
| AAEL015555 | 2.7 | N/A |
| AAEL014026 | 2.7 | C |
| AAEL014026 | 2.7 | C |
| AAEL014026 | 2.7 | C |
| AAEL001918 | 2.6 | N/A |
| AAEL003487 | 2.6 | N/A |
| AAEL004363 | 2.6 | N/A |
| AAEL004408 | 2.6 | N/A |
| AAEL004476 | 2.6 | N/A |
| AAEL004922 | 2.6 | N/A |
| AAEL005555 | 2.6 | N/A |
| AAEL008297 | 2.6 | N/A |
| AAEL009693 | 2.6 | N/A |
| AAEL010712 | 2.6 | N/A |
| AAEL011767 | 2.6 | N/A |
| AAEL011969 | 2.6 | N/A |
| AAEL013106 | 2.6 | N/A |
| AAEL013180 | 2.6 | N/A |
| AAEL015126 | 2.6 | N/A |
| AAEL015383 | 2.6 | N/A |
| AAEL000400 | 2.5 | N/A |
| AAEL000569 | 2.5 | N/A |
| AAEL001386 | 2.5 | N/A |
| AAEL001468 | 2.5 | N/A |
| AAEL001843 | 2.5 | N/A |
| AAEL003979 | 2.5 | N/A |
| AAEL004236 | 2.5 | N/A |
| AAEL004410 | 2.5 | N/A |
| AAEL007884 | 2.5 | N/A |
| AAEL007973 | 2.5 | N/A |
| AAEL009299 | 2.5 | N/A |
| AAEL010898 | 2.5 | N/A |
| AAEL010907 | 2.5 | N/A |
| AAEL011359 | 2.5 | N/A |
| AAEL012085 | 2.5 | N/A |
| AAEL012551 | 2.5 | N/A |
| AAEL013610 | 2.5 | N/A |
| AAEL013634 | 2.5 | N/A |
| AAEL014176 | 2.5 | N/A |
| AAEL014631 | 2.5 | N/A |
| AAEL014696 | 2.5 | N/A |
| AAEL014701 | 2.5 | N/A |
| AAEL015242 | 2.5 | N/A |
| AAEL015339 | 2.5 | N/A |
| AAEL004410 | 2.5 | B |
| AAEL011359 | 2.5 | B |
| AAEL011359 | 2.5 | B |
| AAEL000262 | 2.4 | N/A |
| AAEL000591 | 2.4 | N/A |
| AAEL002336 | 2.4 | N/A |
| AAEL002522 | 2.4 | N/A |
| AAEL002695 | 2.4 | N/A |
| AAEL002802 | 2.4 | N/A |
| AAEL004723 | 2.4 | N/A |
| AAEL005057 | 2.4 | N/A |
| AAEL006878 | 2.4 | N/A |
| AAEL007086 | 2.4 | N/A |
| AAEL007465 | 2.4 | N/A |
| AAEL008091 | 2.4 | N/A |
| AAEL012203 | 2.4 | N/A |
| AAEL012290 | 2.4 | N/A |
| AAEL012428 | 2.4 | N/A |
| AAEL012754 | 2.4 | N/A |
| AAEL012823 | 2.4 | N/A |
| AAEL013459 | 2.4 | N/A |
| AAEL013905 | 2.4 | N/A |
| AAEL014442 | 2.4 | N/A |
| AAEL015145 | 2.4 | N/A |
| AAEL015190 | 2.4 | N/A |
| AAEL012823 | 2.4 | B |
| AAEL000054 | 2.3 | N/A |
| AAEL000324 | 2.3 | N/A |
| AAEL000343 | 2.3 | N/A |
| AAEL000423 | 2.3 | N/A |
| AAEL000727 | 2.3 | N/A |
| AAEL002769 | 2.3 | N/A |
| AAEL002967 | 2.3 | N/A |
| AAEL003901 | 2.3 | N/A |
| AAEL003961 | 2.3 | N/A |
| AAEL004224 | 2.3 | N/A |
| AAEL005074 | 2.3 | N/A |
| AAEL005330 | 2.3 | N/A |
| AAEL005525 | 2.3 | N/A |
| AAEL005832 | 2.3 | N/A |
| AAEL006861 | 2.3 | N/A |
| AAEL007197 | 2.3 | N/A |
| AAEL007531 | 2.3 | N/A |
| AAEL008194 | 2.3 | N/A |
| AAEL008302 | 2.3 | N/A |
| AAEL008362 | 2.3 | N/A |
| AAEL008832 | 2.3 | N/A |
| AAEL009261 | 2.3 | N/A |
| AAEL009400 | 2.3 | N/A |
| AAEL009975 | 2.3 | N/A |
| AAEL011023 | 2.3 | N/A |
| AAEL011138 | 2.3 | N/A |
| AAEL011387 | 2.3 | N/A |
| AAEL011440 | 2.3 | N/A |
| AAEL012747 | 2.3 | N/A |
| AAEL012854 | 2.3 | N/A |
| AAEL012972 | 2.3 | N/A |
| AAEL013575 | 2.3 | N/A |
| AAEL013920 | 2.3 | N/A |
| AAEL014008 | 2.3 | N/A |
| AAEL014851 | 2.3 | N/A |
| AAEL015597 | 2.3 | N/A |
| AAEL008302 | 2.3 | B |
| AAEL012972 | 2.3 | B |
| AAEL013920 | 2.3 | B |
| AAEL008302 | 2.3 | C |
| AAEL005330 | 2.3 | C |
| AAEL003961 | 2.3 | D |
| AAEL003961 | 2.3 | D |
| AAEL003961 | 2.3 | D |
| AAEL003961 | 2.3 | D |
| AAEL003961 | 2.3 | D |
| AAEL003961 | 2.3 | D |
| AAEL003961 | 2.3 | E |
| AAEL012747 | 2.3 | E |
